# Strategies to improve interpersonal communication along the continuum of maternal and newborn care: A scoping review and narrative synthesis

**DOI:** 10.1371/journal.pgph.0002449

**Published:** 2023-10-11

**Authors:** Klaartje M. Olde Loohuis, Bregje C. de Kok, Winter Bruner, Annemoon Jonker, Emmanuella Salia, Özge Tunçalp, Anayda Portela, Hedieh Mehrtash, Diederick E. Grobbee, Emmanuel Srofeneyoh, Kwame Adu-Bonsaffoh, Hannah Brown Amoakoh, Mary Amoakoh-Coleman, Joyce L. Browne

**Affiliations:** 1 Julius Global Health, Julius Centre for Health Sciences and Primary Care, UMC Utrecht, Utrecht University, Utrecht, The Netherlands; 2 Department of Anthropology, University of Amsterdam, Amsterdam, The Netherlands; 3 Department of Genetics, Genomics and Informatics, University of Tennessee Health Science Center, Memphis, TN, United States of America; 4 Department of Sexual and Reproductive Health and Research Including UNDP/UNFPA/UNICEF/WHO/World Bank Special Programme of Research, Development and Research Training in Human Reproduction, World Health Organization, Geneva, Switzerland; 5 Department of Maternal, Newborn, Child and Adolescent Health and Ageing, World Health Organization, Geneva, Switzerland; 6 Department of Obstetrics and Gynecology, Greater Regional Hospital, Accra, Ghana; 7 Department of Obstetrics and Gynecology, University of Ghana Medical School, Accra, Ghana; 8 Department of Epidemiology, Noguchi Memorial Institute for Medical Research, University of Accra, Accra, Ghana; University of California San Francisco (UCSF), UNITED STATES

## Abstract

Effective interpersonal communication is essential to provide respectful and quality maternal and newborn care (MNC). This scoping review mapped, categorized, and analysed strategies implemented to improve interpersonal communication within MNC up to 42 days after birth. Twelve bibliographic databases were searched for quantitative and qualitative studies that evaluated interventions to improve interpersonal communication between health workers and women, their partners or newborns’ families. Eligible studies were published in English between January 1^st^ 2000 and July 1^st^ 2020. In addition, communication studies in reproduction related domains in sexual and reproductive health and rights were included. Data extracted included study design, study population, and details of the communication intervention. Communication strategies were analysed and categorized based on existing conceptualizations of communication goals and interpersonal communication processes. A total of 138 articles were included. These reported on 128 strategies to improve interpersonal communication and were conducted in Europe and North America (n = 85), Sub-Saharan Africa (n = 12), Australia and New Zealand (n = 10), Central and Southern Asia (n = 9), Latin America and the Caribbean (n = 6), Northern Africa and Western Asia (n = 4) and Eastern and South-Eastern Asia (n = 2). Strategies addressed three communication goals: facilitating exchange of information (n = 97), creating a good interpersonal relationship (n = 57), and/or enabling the inclusion of women and partners in the decision making (n = 41). Two main approaches to strengthen interpersonal communication were identified: training health workers (n = 74) and using tools (n = 63). Narrative analysis of these interventions led to an update of an existing communication framework. The categorization of different forms of interpersonal communication strategy can inform the design, implementation and evaluation of communication improvement strategies. While most interventions focused on information provision, incorporating other communication goals (building a relationship, inclusion of women and partners in decision making) could further improve the experience of care for women, their partners and the families of newborns.

## Introduction

Improving the quality of maternal and neonatal health services would accelerate reductions in maternal and neonatal deaths in low- and middle-income countries (LMICs) [1]. Quality of care, as the 2015 World Health Organization (WHO) quality of care framework identifies, has two major domains: provision of care and experience of care [2]. The experience of care dimension includes effective interpersonal communication, which is also closely linked to mistreatment of women during childbirth [3]. In a multi-country study, almost one in five women felt that health workers or staff did not listen and respond to their concerns, and more than half reported no consent for episiotomies performed during childbirth [4].

Effective interpersonal communication is a cornerstone of medical practice [5, 6]. Effective communication can serve three different goals: facilitating the exchange of information, creating a good interpersonal relationship including building of trust, and enabling the inclusion of patients in decision making [7–12]. Communication is furthermore an important theme in respectful maternity care, and a way to protect the human rights of women, for example, through ensuring confidentiality, fulfilling the right to be fully informed and allowing for informed consent [9].

Interpersonal communication between health workers and patients can affect health care outcomes, including patients’ satisfaction, knowledge and understanding, adherence to treatment, quality of life and psychological and physical health [8, 10, 11]. Within maternal and newborn care (MNC), good interpersonal communication contributes to better experiences, improved respectful care and reduced mistreatment [7, 8, 13]. This is particularly relevant in low-resource settings where poor communication and mistreatment are common [4], contributing to negative or traumatic birth experiences [12]. To illustrate, in Kenya, person-centred care, which included many interpersonal communication related aspects, was associated with improved MNC outcomes [13].

Various aspects of interpersonal communication processes between health workers and patients have been described. The communication framework of Feldman-Stewart and Brundage is particularly useful to illustrate ‘how’ interpersonal communication works [14, 15], and can thus be helpful in understanding how interpersonal communication could be improved. First, this framework suggests that both health workers and patients have goals in terms of what they want to achieve during the interaction. Second, each participant has certain needs, beliefs, values, skills and emotions that shape ways to interact. Third, each participant receives and sends messages. And finally, the framework underscores that the environment in which the interaction takes place matters for communication, and thus for strategies designed to improve interpersonal communication [14, 15]. Further guidance on effective communication within MNC is emphasized within WHO’s recommendations across the continuum of MNC [16–18]. While these recommendations do not provide a definition of effective communication within the context of MNC, they provide guidance to ensure effective communication is prioritized between health workers, women, their partners and families [16].

Despite the increased recognition of the importance of interpersonal communication for MNC there is no clear overview of the different strategies that can be adopted to reduce mistreatment and improve respectful care [19]. Therefore, the objective of this review was to map and categorize implemented strategies to improve interpersonal communication between health workers, women and their partners within MNC up to 42 days after birth.

## Methods

### Protocol and registration

This review was drafted and conducted in accordance with the PRISMA guidelines [20] and the Cochrane Handbook for Systematic Reviews [21]. The study protocol was registered in PROSPERO in July 2020 (CRD42020191622). The protocol was initially developed for a systematic review including a possible meta-analysis on effectiveness. We converted to a scoping review with narrative analysis due to the number and heterogeneous nature of the primary research articles, and because this provided a sufficient basis for answering the research questions.

### Domain and population

The domain of our review consisted of studies that implemented a strategy to improve interpersonal communication between health workers, women, and their partners in care across the continuum of MNC. We also anticipated that experiences to improve interpersonal communication from related domains in sexual and reproductive health and rights (SRHR) would facilitate cross-learning from MNC, and therefore extended the domain to include the reproduction-related SRHR subdomains of safe abortion, family planning and (in)fertility.

The study population included women and their partners as well as newborns and their parents/caregivers/families throughout the continuum of MNC and reproduction-related SRHR subdomains. This included antenatal, intrapartum and postnatal care up to 42 days. In this paper, we used ‘women and partners’ to describe the population. Health workers included different cadres as specified in the WHO recommendations to optimize health workers’ roles within MNC [22]: lay health workers, (auxiliary) nurses and midwives, and (associate) physicians (including obstetricians, paediatricians, general practitioners and residents).

### Eligibility criteria

Studies were eligible for inclusion if they were primary, peer-reviewed articles reporting on interpersonal communication quality improvement strategies between health workers and women and partners in MNC and reproductive-related SRHR subdomains. All studies that included health workers who were engaged with in-service training (i.e., not training by students as part of a qualifying degree) were eligible. Studies conducted in any setting within MNC and reproductive-related SRHR subdomains were eligible. Studies that included a paediatric population were only eligible if >50% of participants were newborns (up to 42 days old) or newborns’ parents. Studies published from January 2000 to July 2020 were included to reflect contemporary practices.

We excluded studies that focused on mass communication, group communication, one-way communication, interprofessional communication and communication between mothers and babies. Furthermore, we excluded studies that reported on packages of strategies where communication was not a primary aim, because in these complex intervention studies communication was usually a small part of the intervention, and so difficult to disentangle from other activities. Furthermore, studies that implemented a new communication-based treatment programme (e.g., cognitive behaviour therapy) to treat a specific disease or problem were excluded because these constituted a new form of health service delivery, except when the intervention specifically focused on improving the *interpersonal communication* within the delivery of the health service. We excluded reviews, but primary studies from relevant systematic reviews were checked for eligibility. We included only English articles, because of language limitations within the team. In total, six studies were excluded in full text screening because of language restrictions.

### Information sources

We searched the following information sources: PubMed/Medline, EMBASE, CINAHL, SCOPUS, PsychINFO, Anthropology PLUS, SocioINdex, Cochrane Central Register of Controlled Trials (CENTRAL), Latin American and Caribbean Health Sciences Literature (LILACS), African Journals Online (AJOL), and Global Health Library.

### Search

Search terms consisted of MeSH and combined text related to ‘communication’, ‘health workers’, ‘MNC or related SRHR domains’, ‘women and families’ and ‘intervention’. The search was developed with support from a librarian. For the complete search strategy see S1 Appendix. References of included articles were snowballed and checked for eligibility. De-duplication was performed using Endnote (V.X9).

### Selection of sources of evidence

First, titles and/or abstracts of studies identified through the search strategy were independently screened to assess whether studies met the inclusion criteria by two of the four reviewers (AJ, ES, WB, KMOL). Next, full texts were screened in the same way. In case a full text article was missing or inaccessible, authors were contacted once through email or ResearchGate and were given the option to respond within a month to provide us with the full text. Rayyan QCRI (https://rayyan.qcri.org/welcome) was used to screen articles. Any disagreement that arose was discussed by the persons who screened the articles until consensus was reached, or a fifth review team member was consulted (JLB or BCdK) to resolve the issue through further discussion.

### Data charting process including data items

Data were extracted using a standardized pre-piloted form (by AJ, ES, WB, KMOL, BCdK). The data extracted included study characteristics and information for evidence synthesis: first author, year of publication, country, study setting, aims and objectives, study design, study population characteristics, description of intervention and communication goals of the intervention, and the types of outcomes measured. Extracted data were double checked by one of the team members (KMOL).

### Synthesis of results

We initially planned to perform a systematic review including a meta-analysis on interpersonal communication strategies’ effectiveness. However, the number and heterogeneity of designs and interventions among the retrieved articles led us to convert the study to a scoping review with narrative synthesis that focussed on providing an overview and categorization of the various strategies taken to improve interpersonal communication [23]. In this scoping review process, we summarized key findings of articles. We categorized strategies into the three communication goals proposed by Ong et al. [8]: 1) to facilitate the exchange of information, 2) create a good interpersonal relationship, and 3) enable the inclusion of women and partners in the decision making. These categories were pragmatically used as heuristic tools, i.e. functional methods (not necessarily perfect), and studies were assigned to one or more of these categories based on the information available. In addition, we analysed the results to understand ‘how’ interpersonal communication and the communication improvement strategies worked, using the model proposed by Feldman-Stewart and Brundage [14]. This model was updated (and re-visualized) with insights from this review and further deliberations within the review team.

## Results

**Fig 1:**
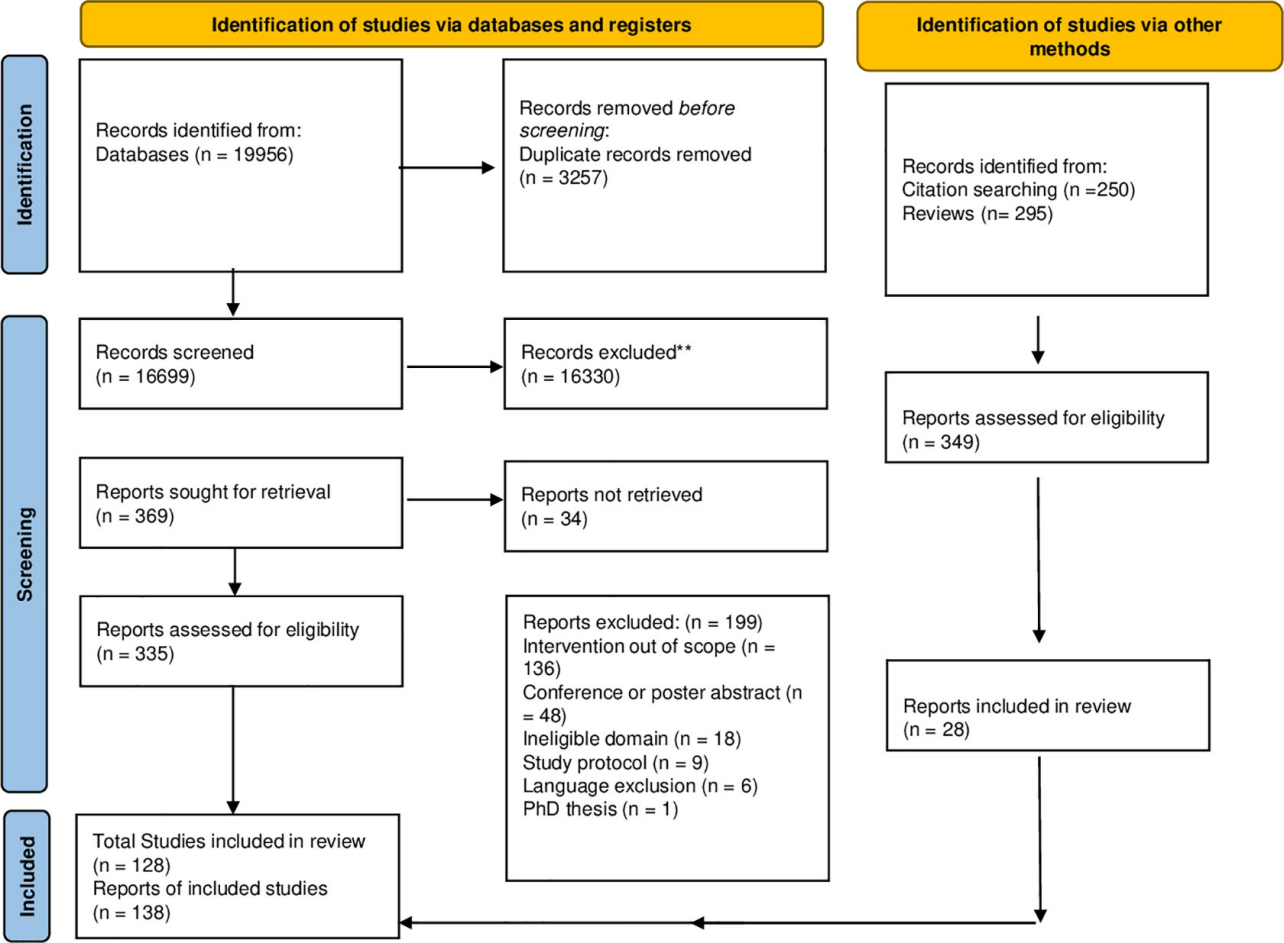
PRISMA flow diagram of included studies.

We identified a total of 19956 articles through our search (see flow diagram in Fig 1). After removing duplicates, we screened 16826 articles on title and abstract and 369 articles in full text. Twenty-nine articles were additionally included through snowballing and reference screening of review articles. A total of 138 articles were included, reporting on 128 strategies to improve interpersonal communication.

### Study characteristics

Table 1 presents an overview of the included studies. Interventions were implemented in Europe and North America (n = 85), Sub-Saharan Africa (n = 12), Australia and New Zealand (n = 10), Central and Southern Asia (n = 9), Latin America and the Caribbean (n = 6), Northern Africa and Western Asia (n = 4) and Eastern and South-Eastern Asia (n = 2). The majority were in high-income countries (n = 95), compared to 33 interventions in LMICs [24]. The majority of studies were performed within the domain of maternal health (n = 80 studies), with others performed in newborn health (n = 47), family planning (n = 20) and (in)fertility (n = 2). Thirty-seven were randomized controlled trials (RCTs), 73 were non-RCT intervention studies.

**Table 1:** Overview of included studies.

Author and year	Setting	Study Design	Participants	Domain	Description of the intervention	Type of intervention	Communication goal	Key findings Visual summary of direction of effect (green = positive effect, orange = no effect, red = negative effect. Yellow = mixed outcomes
Afulani et al. 2019	Ghana, referral hospital and health centres	Non-RCT	Women who gave birth at the facility, pre-implementation n = 268 and post-implementation n = 320. Target group: midwives, medical doctors, anestesiologist, nurses (n = 43).	Maternal Health	A 2.5 day simulation training with case-based scenarios about dignity and respect, communication, respecting autonomy, and supporting them in whichever way women needed including encouraging birth companions.	Training	Creating relationship and inclusion of women in decision making	Higher respectful maternity care measurement in intervention group.
Ahmadi et al. 2016	Iran, NICU centre	RCT	Mothers with premature infants, intervention n = 62, control n = 62.	Newborn Health	Counseling of women about exclusive breastfeeding using a predefined model (GATHER counselling steps).	Tool	Exchange of information and creating relationship	The intervention group had significantly higher rates of exclusive breastfeeding.
Alder et al. 2007	Switzerland, OBGYN department at a university hospital	RCT	Primary care physicians providing antenal care (also target group), intervention n = 16, control n = 16. Pregnant women, n = 24.	Maternal Health	A 1-day workshop on communication and interpersonal relationships, combined with three half day practice seminars and 5 or 6 one-hour supervisions.	Training	Creating relationship and inclusion of women in decision making	There was no difference found within observer-based ratings of communicative skills between training and control group. There was an increase in patient satisfaction for patients of the training group.
Andaroon et al. 2020	Iran, different health-care centres	RCT	Nulliparous women, intervention n = 45, control n = 45.	Maternal Health	Counselling session about giving birth using a framework (BELIEF counselling model).	Tool	Exchange of information and creating relationship	The choice for natural childbirth in nulliparous women at 34–36 weeks of pregnancy in the intervention group was significantly higher than in the control group.
Antoniou et al. 2012	Canada, clinic	RCT	Anesthesia residents, intervention n = 10, control n = 10.	Maternal Health	Training with simulation (intervention) versus normal didactic session on informed consent (control).	Training	Exchange of information	Both groups showed significant improvement in their written consent documentation at the immediate time point, the improvement in the didactic group being greater. The didactic group performed better at both the immediate time point and the six-week time point.
Arimori 2006	Japan, general hospital	RCT	Prenatal women, intervention n = 48, control n = 48. Intervention conducted by nurse.	Maternal Health	Structured decision aid guide combined with genetic counselling.	Tool	Exchange of information and inclusion of women in decision making	There were no changes in decisional conflict scores.
Baijens et al. 2018	Netherlands, academic hospital	Non-RCT	Obstetric inpatients of the obstetric ward of a university hospital, with complex obstetrical problems, pre-intervention n = 35, post-interventio n = 29, interviews n = 4.	Maternal Health	Patients received a card with three questions (prompt) stimulating Shared Decision Making, used during ward rounds.	Tool	Inclusion of women in decicion making	No difference in shared decision making score in pre- and post-intervention group. In the interviews, patients reported the three questions to be very useful. They used the questions mainly as a prompt and encouragement to ask more specific questions.
Baird et al. 2018	Australia, hospitals	Non-RCT	Midwifes and nurses, n = 149.	Maternal Health	Full-day simulation based training about discussing sensitive topics (domestic violence) in pregnancy.	Training	Exchange of information and creating relationship	Increased knowledge and preparedness to discuss sensitive topics.
Baird et al. 2018 *secondary article beloning to Baird 2018		Non-RCT	Midwifes offering antenatal care, n = 83.					Knowledge and preparedness increased after training, at 6 months follow up self reported practice improvements.
Bakker et al. 2003	Netherlands, midwife community practices	Post implementation evaluation study	Midwifes, intervention n = 57, control n = 61.	Maternal Health	Brief manual, intervention card and training on a counselling protocol/method on smoking cessation in pregnancy. Protocol consisted of 7 steps. Visual aid included.	Training and tool	Exchange of information	Increased frequency of giving advise to quit smoking, setting a date, and providing aftercare.
Bashour et al. 2013	Syria, tertiary care teaching maternity hospitals	RCT	OBGYN residents, n = 137.	Maternal Health	Training effective communication skills, focus on interaction.	Training	Creating relationship	No increase in patient satisfaction, no significant improvement of observed communication.
Bekker et al. 2004	UK, prenatal diagnosis clinics	RCT	Women who received positive maternal serum screening result for Down’s syndrome risk. Intervention n = 59, control n = 58.	Maternal Health	Prompts to facilitate decision analysis to improve decision making about prenatal diagnoses for Down’s syndrome	Tool	Exchange of information	Decision consultations helped women to make more informed decisions about prenatal diagnosis. Informed decision making was increased and women perceived the risk to be more realistic. Furthermore, a decrease in decisional conflict in the intervention (decision analysis) group. Decision analysis had no impact on knowledge or SEU scores, directiveness or usefullness compared to routine care. It did not affect anxiety provocation. On average, consultations took e six minutes longer
Biasini et al. 2012	Italy, pediatric and neonatal intensive care unit	Non-RCT	Families of newborns in NICU (n = 8).	Newborn Health	A communication strategy with three stages: (1) Training in Communication, (2) Communicative Algorithm with various guidelines to follow during the most common scenarios in the NICU, (3) Communicative Case Sheet, a notebook used to record any problem or discomfort that occurs during communication.	Tool, Training	Exchange of information and creating relationship	75% of participants scored satisfaction with communication as very good; among the remaining 25%, parents perceived communication as good, but improvable.
Boss et al. 2013	USA, academic hospital, department of neonatology	Non-RCT	NICU fellows and neonatal nurse practitioners (NNPs). Intervention n = 13, control, n = 13.	Newborn Health	3-day communication skills training with focus on communicating bad news, settings goals of care and addressing conflict. Part of the training simulation training in small groups.	Training	Exchange of information	Increased feeling of preparedness and competence of participants after the training to deal with difficult conversations.
Boss et al. 2012 *secondary article belonging to Boss et al. 2013		Non-RCT	Neonatalogy physician (n = 6) and neonatology fellows (n = 4).					Physicians found the simulation training realistic. They spent most time on biomedical information provision, and 20% on building relationships.
Bowen et al. 2020	USA, tertiary medical Centre	RCT	NICU nurses, intervention n = 5, control = 7.	Newborn Health	A 4.5 hour ’Difficult Conversations’ workshop including simulation.	Training	Exchange of information and creating relationship	Improved predefined communication skill behaviours after intervention. Mean empathy score was significantly higher in the intervention group. Participant satisfaction was high.
Brasington et al. 2016	Egypt, communities	Non-RCT	Pregnant women or mothers with a child under age of 2 years. Intervention n = 1602, control n = 1597. Target: community health workers (CHWs).	Maternal and Newborn Health	Training on health communication including role plays.	Training	Exchange of information	Post-intervention knowledge regarding care seeking for danger signs improved significantly, improved antenatal care seeking behaviour. There were no improved breastfeeding practices and number of women who received skilled birth attendance.
Bry et al. 2016	Sweden, NICU of university hospital	Non-RCT	NICU nurses (target, n = 13) and parents (pre-intervention n = 26, post-intervention n = 30).	Newborn Health	A 2-hour lecture and 1-day workshop on parents’ needs, communication skills, emotional support, identifying parental emotions.	Training	Creating relationship	The ratio nurse/patient spoken words decreased, increased empathic responses, exploring parental feelings increased. Nurses: increased awareness about the need, increased confidence and ways to express empathy.
Calderon et al. 2008	USA, prenatal clinics	RCT	Pregnant women receiving prenatal care. Target group: providers.	Maternal Health	Summary "cueing sheet" (prompt) and guidance on counselling for the provider.	Tool	Exchange of information	Increased reporting of discussions with provider about intimate partner violence.
Chin-Quee et al. 2007	Nicaragua, different family planning clinics	Non-RCT	New users of family planning methods, intervention sites n = 41, control sites n = 24.	Family Planning	DMT flipchart decision aid tool.	Tool	Exchange of information and inclusion of women in decision making	Experience of care increased, no difference of anticonception continuation rates between groups.
Chinkam et al. 2016	USA, academic medical center	Non-RCT	Pregnant women, intervention n = 22, control n = 22.	Maternal Health	Scripted counseling package about birth choices and trial of labour after cesarean, using shared decision principles.	Tool	Exchange of information and inclusion of women in decision making	Increased number of participants who believed they had enough information after the intervention.
Chor et al. 2020	USA, abortion clinics	Non-RCT	Women presenting for abortion who lacked a regular health care provider and desired to delay pregnancy for at least 6 months (n = 60). Target group: lay healthcare workers (LHW).	Safe Abortion	Training on behavioral theory–based counselling.	Training	Exchange of information	Participants felt comfortable speaking to their LHW. They appreciated the supportive, approachable LHWs, talking through options, and setting goals.
Chuffo Siewert et al. 2015	USA, NICU	Non-RCT	New mothers with postpartum depression. Target group: nurses.	Maternal Health	Listening Visits, a structured method to listen to mothers and let them lead the conversation.	Tool	Creating relationship and inclusion of women in decicion making	Intervention group associated with reduction in maternal depressive and anxiety symptoms.
Clarke-Pounder et al. 2015	USA, tertiary NICU	RCT	Parents and providers, intervention n = 9, control n = 10.	Newborn Health	Adapted version of the decision-making tool (N-DMT) made for NICU parents consisting of 4 areas: 1) medical indications for treatment, 2) parent preferences, 3) quality of life, and 4) contextual issues. Goal was to encourage parents to share needs and values relevant to medical decision making.	Tool	Exchange of information and inclusion of women in decision making	Families who used adapted N-DMT were less satisfied with communication than control families. The control group showed decreased rates of anxiety, the intervention group did not. Baseline anxiety levels were not assessed.
Dehlendorf et al. 2019	USA, safety net primary care clinic	RCT	Providers (n = 15).	Family Planning	Decicion support tool (*my-birth control*) to facilitate shared decicion making for women and their providers.	Tool	Exchange of information and inclusion of women in decision making	No differences in length of conversations. Patients who used My Birth Control were more confident in describing their method preferences. Physicians reported that incorporating My Birth Control into their counselling practice was both acceptable and feasible, no differences in Burnout inventory.
Dehlendorf et al. 2017 *secondary article beloning to Dehlendorf et al. 2019		Non-RCT	Female patients at safety net clinics. Intervention n = 42, control n = 41.					Supportive for method choosing (96%), qualitative interviews indicated acceptability of the tool’s content and presentation.
Guillén et al. 2019	USA, NICU	RCT	Women facing extreme premature delivery. Intervention n = 99, control n = 102. Target group: clinicians.	Maternal and Newborn Health	Decision-aids to explain options, quantify potential risks and benefits, and provide structured guidance in deliberation and communication for providers and parents expecting a premature infant.	Tool	Exchange of information and inclusion of women in decision making	Both groups decisional conflict scores were low and preparedness for decision-making scores high. Significantly improved knowledge of complex information. Clinicians found it was helpful in understanding risk and making decisions.
Guilleén et al. 2012 *secondary article belonging to Guillén et al. 2019	USA, hospital	Non-RCT	Clinicians (n = 31) (neonatologists, neonatal fellows, neonatal nurses, and maternal-fetal medicine specialists), and parents of infants born before 26 weeks’ gestation (n = 30).	Newborn Health				Significant improvement in knowledge after intervention.
de Jersey et al. 2018	Australia, tertiary hospitals	Non-RCT	Midwives, pre-intervention n = 154, post-intervention n = 114.	Maternal Health	A 40-minute training session on information provision and counselling for healthy weight during pregnancy using 5A counselling framework.	Training	Exchange of information	Increased perceived and objective knowledge of participants. Increased perceived confidence of participants
Dormandy et al. 2012	UK, general practices	RCT	Health care professionals, intervention n = 64, control n = 76.	Maternal Health	Simulation training on general communication skills.	Training	Exchange of information	More frequent screening and at an earlier gestational age in intervention group.
Drago 2018	USA, academic follow-up clinics	Post implementation evaluation study	“Experienced” parents (non-pregnant Latino parents with a history of premature birth <26 weeks, n = 9) and “naive” parents (non-pregnant Latino adults, >18 years of age, no prior history of premature birth, n = 10).	Newborn health	Adapted decision aid tool for Latino parents facing extreme premature delivery.	Tool	Exchange of information	Increased knowledge scores among ’naïve’ women, ‘Experienced’ parents had a ceiling effect. The tool was well received by participants.
Dulmen & Weert 2001	The Netherlands, hospital	Non-RCT	OBGYNs, n = 18.	Maternal Health	Training sessions on different topics: education on the importance of a therapeutic relationship, of communication affect (non-verbal and verbal), addressing psychosocial issues, and of giving the patient time to talk. The training further included role playing excercises, feedback sessions, discussions with peers.	Training	Exchange of information and creating relationship	As a result of the training, gynaecologists’ sensitivity to psychosocial aspects increased. They gave more signs of agreement, became less directive, asked fewer medical questions and more psychosocial questions. No difference was found regarding the duration of the outpatient visits. With the trained gynaecologists, patients asked more questions and provided more psychosocial information.
Ekström et al. 2012	Sweden, prenatal and child health centres	RCT	Primiparous pregnant women, intervention n = 172, control n = 308. Target group: prenatal midwives, postnatal nurses and others.	Maternal Health	A process-oriented training programme on breastfeeding counselling. Elements were evidence-based information, reflective processes, professional stance, problem-solving processes and how to provide support.	Training	Exchange of information and creating relationship	In the intervention group, mothers had a significantly longer duration of exclusive breastfeeding.
Everett-Murphy et al. 2010	South Africa, public sector antenatal clinics	Non-RCT	Pregnant women of low socio-economic status and smoking. Intervention n = 536, control n = 443.	Maternal Health	Midwives were trained to use the ACOG 5As Guideline for brief smoking cessation counselling method including motivational interviewing principles.	Training and tool	Exchange of information and creating relationship	Increased quitting rates and reduction of smoking in intervention group.
Farnworth et al. 2008	UK, infirmary	Non-RCT	Women with a history of one previous caesarean section and no previous vaginal deliveries. Intervention n = 16, control n = 16.	Maternal Health	Informational video, visual aid before counselling session at home.	Tool	Exchange of information	Similar knowledge about risks and benefits in both study groups. Interviews revealed that rather than being passive recipients of information, women who received the face-to-face intervention felt more confident in their choices.
Farrell et al. 2014	USA, different settings	RCT	Family medicine physicians or pediatricians.	Newborn Health	Assesment of communication with feedback using rapid-throughput report cards, during two phone consultations with standardized patient.	Training	Exchange of information and creating relationship	Improved communication behaviours, including: request for teach-back, opening behaviours, anticipation or validation of emotion and the ratio of explained to unexplained jargon words.
Farrokh-Eslamlou et al. 2014	Iran, 52 urban and rural public health facilities	Non-RCT	Women visiting family planning clinic, pre-intervention n = 448, post-intervention n = 547. Target group: service providers, n = 78.	Family Planning	Use of the WHO decision-making tool for family planning, including 2 days of training.	Tool	Exchange of information and inclusion of women in decision making	More active participation of clients in post-intervention group, informed choice increased. Informed information provision including technical competenties. Improved client-provider interpersonal interactions and increased proportion of sessions in which counselling resulted in a choice of a contraceptive method. Higher patient satisfaction.
Fatima et al. 2018	Bangladesh, tertiary hospitals	Non-RCT	Women delivering in hospital (pre-implementation n = 11,263, post-implementation n = 16,359. Target group: family planning counselers, n = 28.	Family Planning	4-day training on counselling skills based on GATHER (Greet, Ask, Tell, Help, Explain, Return) and factual information on contraception and postpartum intrauterine devices (PPIUD).	Training	Exchange of information	Increased understanding of counsellors, increased proportion of women who were counselled, decrease in proportion of women agreeing with postpartum contraceptive method. No impact on overall PPIUD insertion rate.
Fay 2016	USA, newborn care unit at academic hospital	Non-RCT	Parents (n = 32) and clinicians (n = 5).	Newborn Health	Option Grud tool (1-page document about treatment options) and a training session, focused on facilitating discussion and eliciting parental preferences.	Training and tool	Inclusion of women in decision making	Higher OPTION (shared decicion making) scores post-intervention.
Fenwick et al. 2013	Australia, setting not further specified	Post implementation evaluation study (qualitative)	Postpartum women who experienced a traumatic birth. Intervention n = 16, active control n = 12, standard care n = 5.	Maternal Health	Structured counselling intervention, key elements: therapeutic connection, acceptence and work with women’s perceptions, encourage expression of feelings, filling missing pieces, connect the event with emotions and behaviours, review the labour management, enhance social support, reinforce positive approaches to coping, explore solutions.	Tool	Creating relationship	Women in all three groups felt being cared for, and felt they were promoted to reflect. Women in PRIME intervention felt they got in touch with their feelings, and felt they could ’move on’. Some women in both groups felt that the contact was nice, but not heavily needed.
Fenwick et al. 2018	USA, maternity department of hospital	Non-RCT	Midwives, n = 19.	Maternal Health	3 half-day training sessions for providers (including simulation) about relationships and micro-counselling, psycho-education.	Training	Exchange of information and creating relationship	Significant improvement of midwives’ knowledge, skills and confidence to counsel women on psychosocial issues and reduce fear scores for women reporting high childbirth fear.
Figueroa et al. 2020	USA, family medicine practices	Non-RCT	Pregnant women (pre-implementation n = 795, post-implementation n = 776). Intervention target group: support staff, nurses, residents, faculty, and teaching attending physicians.	Maternal Health	6 months’ counselling training with focus on motivational interviewing techniques, work protocols, tools (ehealth prompts, handouts).	Training and tool	All three goals	Increased number of women who received counselling. Small difference in prevalence of gestational diabetes.
Gamazina et al. 2009	Ukraine, maternity centres, health facility, hospital	Non-RCT	OBGYNs and midwives providing counseling about prevention of mother-to-child-transmission HIV (n = 290).	Maternal Health	Training to develop behaviour change, and use of communication materials (visual aids) for pregnant women and families with HIV.	Training and tool	Exchange of information	Consistently higher quality prevention of mother to child transmission counselling among training participants.
García et al. 2013	Spain, private fertility clinic	Non-RCT	Patients receiving consultations at the clinic, n = 2146. Target group: fertility physicians, n = 13.	(In)Fertility	2-day training of physicians with a focus on empathy	Training	Creating relationship	Higher scores of satisfaction in all domains: information, dynamic, time and rhythm, interaction and professionalism.
Gerancher et al. 2000	USA, hospital	RCT	Women presenting in labour, intervention n = 44, control n = 38.	Maternal Health	A ten-points informed consent form covering topics that were discussed during the informed consent procedure to make sure women understood the information well.	Tool	Exchange of information	Recall score improvement in the informed consent and written group.
Glavin et al. 2010	Norway, municipality	Non-RCT	Postpartum women, intervention n = 164, control n = 64. Target group: public health nurses (n = 26).	Maternal Health	Training about postpartum depression and supportive counseling using active listening and empathic communication. Monthly supervision of nurses.	Training	Exchange of information and creating relationship	Lower depression score in the intervention group at 3 and 6 months postpartum.
Guillén et al. 2016	USA, tertiary care hospital	Post implementation evaluation study	Clinicians (n = 31), parents of extremely premature infants (n = 14) and "naive" healthy, non-pregnant women with no history of premature delivery (n = 13).	Newborn Health	Counselling session supplemented by a 10-minute video decision aid about extreme prematurity.	Tool	Exchange of information	Well accepted video by parents and "naive" parents. Message was perceived as ’balanced and neutral’.
Gunn et al. 2006	Australia, public tertiary hospital	Non-RCT	Medical staff, pre/post intervention group midwifes (n = 8) and medical practitioners (n = 4).	Maternal Health	Communication training about women-centred care.	Training	Creating relationship	Higher reported confidence, competence and knowledge to deal with psychosocial issues.
Hajarian Abhari et al. 2020	Iran, health care centres	RCT	Pregant women with basic literacy levels, intervention group n = 30, control group n = 30. Intervention delivered by OBGYN.	Maternal Health	Counseling sessions based on Gamble’s approach (preventive strategy including; building relationship, acceptence of maternal perceptions towards labour, supporting women in expressing emotions etc) to reduce traumatic birth experiences	Tool	Exchange of information and creating relationship	Lower scores of birth trauma in intervention group.
Hall et al. 2019	USA, NICU departments, hospitals	Non-RCT	NICU staff, intervention n = 66, control n = 66.	Newborn Health	7-hour learning modules that included demonstrating simulated conversations between NICU staff and parents. Topics: "(1) communication skills, (2) providing emotional support to parents, (3) peer-to-peer support, (4) family-centred developmental care, (5) palliative and bereavement care, (6) discharge and follow-up support, and (7) supporting staff as they support families."	Training	Creating relationship	After taking the course, there was significant improvement in knowledge and attitudes by the NICU staff in all modules.
Hegarty et al. 2007	Australia, tertiary academic hospital	Non-RCT	Midwifes (n = 21) and doctors (n = 5). Pregnant women pre-training survey n = 584, pregnant women post-training survey n = 481.	Maternal Health	4-interactive workshops with simulation about discussing psychosocial problems. Focus: understanding woman’s experience of pregnacy, finding common ground, information provision. This was followed by additional sessions every 2 weeks over 26 weeks.	Training	Exchange of information and creating relationship	Post-training, women were more likely to report professionals asking them questions about psychosocial wellbeing and women felt more confident discussing these issues.
Henrikson et al. 2015	USA, pediatric and family practice outpatient clinics	RCT	Mothers of healthy newborns (intervention n = 242, control n = 246), physicians (intervention n = 257, control n = 206).	Newborn Health	45-minutes training session on communication related to vaccination hesitancy, importance of building trust.	Training	Creating relationship	The intervention had no detectable effect on maternal vaccine hesitancy; physician self-efficacy in communicating with parents was not significantly different between intervention and control group.
Jennings et al. 2011	Benin, public maternities	Non-RCT	Pregnant women. Target group intervention: lay nurse aides (n = 203). Control group: nurse-midwifes (n = 206).	Maternal Health	Training of lay-nurse aides on communication skills and the use of job-aids so that task shifting could take place.	Training and tool	Exchange of information	Lay nurse aides provided effective antenatal counselling in maternal and newborn care in facility-based settings.
Jennings et al. 2010	Benin, public health facilities	RCT	55 providers, intervention arm n = 26, control arm n = 29. Total of 211 observed consultations in baseline intervention arm, 204 in endline intervention arm; 119 in baseline control arm, 152 in endline control arm.	Maternal Health	Counselling job aids consisting of a set of pictorial counselling cards with culturally appropriate images, training and implementation support.	Training and tool	Exchange of information	Provision of recommended messages to pregnant women significantly improved in the intervention arm compared to the control arm in birth preparedness, danger sign recognition, clean delivery, and newborn care. Significant gains were observed in the mean percentage of communication techniques applied and duration (minutes) of antenatal consultations. The proportion of pregnant women with correct knowledge also significantly improved for multiple topics. Job aids were perceived as positive by providers and pregnant women.
Jennings et al. 2015 *Secondary article nested in Jennings 2010 study		RCT	Recently delivered women and newborn pairs. Baseline intervention n = 95, endline intervention n = 161; baseline control cohort n = 56, endline control cohort n = 99.					Recommended messages provided to recently-delivered women significantly improved in the intervention arm. Proportion of newborns thermally protected within the first hour and delayed for bathing significantly increased. No significant changes were observed in early breastfeeding. The proportion of mothers with correct knowledge of maternal and newborn danger signs grew, as did awareness of several practices about home care.
Johnson et al. 2010	Nicaragua, Mexico and Indonesia, different health facilities	Non-RCT	Women/clients who came for a family planning counseling sessions. N = 426 in Nicaragua, n = 83 in Mexico, n = 96 in Indonesia). Target group: providers (doctors, nurses, nurse assistants).	Family Planning	WHO decision aid tool, including a 2–4 day workshop.	Tool	Exchange of information and inclusion of women in decision making	Use of the tool improved providers’ counselling performance: more engagement with better and tailored information. Increased communication and involvement for clients. Both the Nicaraguan and Mexican studies found marked shifts toward the client in the locus of decision-making.
Kim et al. 2007	Nicaragua, 49 government health facilities	Non-RCT	Service providers, including doctors, nurses, nurse assistants (n = 59).	Family Planning				Increased decision-making score of providers. Improved aspects reported for: responding to needs, efforts to involve clients in decision-making process, better screening for and educating about method. Better decision-making scores by clients were associated with higher likelihood to leave with preferred method, larger impact observed on behaviour of lower educated clients.
Kakkilaya et al. 2011	USA, university hospital	RCT	Pregnant women, intervention n = 44, control n = 45. Intervention target: neonatology fellow.	Maternal and Newborn Health	Counselling using a visual aid with visual/graphical information for parents when delivery at the threshold of viability was imminent.	Tool	Exchange of information	Improvement of mothers knowledge (understanding and recall).
Kasat et al. 2018	USA, NICU department	Non-RCT	NICU providers (intervention n = 109) and parents (pre-intervention n = 127, post-intervention n = 123).	Newborn Health	2-hour workshop on empathy. Focus on empathy skills, breaking bad news, non- verbal communication, helping with compassion.	Training	Creating relationship	Overall, no difference in parent surveys pre and post-intervention. Improvement on specific topics observed: referring to babies name, and being offered emotional support. Providers felt they were more comfortable with communication including difficult conversations and end of life issues.
Kim et al. 2005	Mexico, various government health facilities	Non-RCT	Family planning providers, n = 13.	Family Planning	WHO decision-making tool for family planning	Tool	Exchange of information and inclusion of women in decision making	After tool implementation, more information provision, more tailored to patients and there was more inclusion of patients in decision making. Clients reported that the tool helped them understand the provider’s explanations and made them feel more comfortable talking and asking questions during consultations.
La Rosa et al. 2018	USA, University Hospital	Non-RCT	Postpartum women, intervention n = 89, control n = 93. Intervention target group: postpartum physicians.	Maternal Health	Wearing a whitecoat.	Other	Creating relationship	No differences in reported communication skills.
Langston et al. 2010	USA, family planning referral clinic	RCT	Women seeking a first trimester procedure for a spontaneous or induced abortion. Intervention n = 114, control n = 108. Target providers: faculty, fellows, residents.	Family Planning	Structured counselling session using decision support tool from WHO.	Tool	Exchange of information and inclusion of women in decision making	No difference in choosing very effective method in the intervention group compared to the control group; no difference in starting immediately with anticonception use.
Lechner et al. 2016	USA, department of pediatrics tertiary centre	Post-implementation evaluation study	Graduates of the neonatal-perinatal medicine fellowship, n = 28.	Newborn Health	Two or three 5-to 8-hour workshops with simulation scenarios to improve communication.	Training	Creating relationship	Decrease in percentage of fellows who felt lack of confidence about difficult conversation communication; increased level of comfort delivering bad news and increased specific communication skills.
Lemani et al. 2017	Malawi, primary care facility	RCT	Health surveillance assistants (n = 15 both intervention and control group), and sexually active women (intervention group n = 430, control group n = 387).	Family Planning	2-days of training on family planning counselling for couples.	Training	Exchange of information	No differences in groups towards modern method use (high usage both groups).
Lemmon et al. 2018	USA, NICU unit	Non-RCT	Parents (n = 10) and clinicians (n = 10).	Newborn Health	A question prompt list to guide parents in their communication with the NICU unit staff.	Tool	Exchange of information	Providers and clinicians found the content of the list acceptable. Parents found the list useful and helpful in preparations of meetings with health care team of their baby.
León 2003	Peru and Guatemala, the Peruvian national family planning program and Guatamalan health centres and rural health posts.	Non-RCT	Peru: 25 providers in first workshop, 75 family planning coordinators in second workshop, and 278 providers in second training. Guatamala: 320 providers.	Family Planning	Two 2-day workshops on using a specific method of counselling with the use of different job aids.	Training and tool	Exchange of information and inclusion of women in decision making	Peru: quality of care improved, especially with those providers who used the job aids. Mixed effects on clients knowledge. Guatemala: quality of care improved, most providers used the job aids, time used to counsel increased.
Leshabari et al. 2007	Tanzania, healthcare site	Non-RCT	Mothers who gave birth, intervention site n = 30, comparison site n = 29. Target group: counsellors.	Newborn Health	Integrated programme of counsellor job aids, mother take-home materials (visual aids) and health professional training.	Training and tool	Exchange of information	Study was found to be easily implemented. High satisfaction from both mothers and counsellors, increased counsellor knowledge, increased knowledge of mothers.
Lindberg et al. 2014	USA, ambulatory clinics: two obstetrics clinics, two family practice clinics, and one certified nurse midwife clinic	Non-RCT	Pregnant women receiving antenatal care, n = 388 pre-intervention, n = 345 post-intervention. Target group: obstetricians, family practice physicians, and certified nurse midwives.	Maternal Health	Educational alerts (prompt) combining personalised information about risks/weight with a tool/example on how to counsel and individualised patient information sheet.	Tool	Exchange of information	Rate of weight gain counselling improved, including an increase of counselling according to guidelines.
Lindhardt et al. 2014	Denmark, setting not further specified	Non-RCT	Obstetric healthcare providers working with obese pregnant women (n = 11).	Maternal Health	Training in the use of a motivational interview technique.	Training	Exchange of information and creating relationship	In general, the participants changed their behaviour according to the motivational interviewing technique: they made more interventions related to motivational interviewing principles, they asked fewer closed and more open questions. Most of the participants scored higher after the training in motivational interviewing. Empathy scores did not differ significantly before and after training.
Lobatch et al. 2019	USA, metropolitan hospital	Non-RCT	Mothers being admitted to mother-baby unit (pre-intervention n = 94, post-intervention n = 61).	Maternal Health	Training, combining didactic sessions with role-play on rounding communication skills.	Training	Exchange of information	No significant change in women’s perceptions of nursing care and communication was found between pre- and post-intervention groups. Women were less likely to recommend the hospital during the post-intervention period than in the pre-intervention period.
Macdonell et al. 2015	Canada, NICU	Non-RCT	Staff members of NICU (n = 40), parents of preterm infants (n = unknown).	Newborn Health	Regular in-service training of one hour to stimulate discussion on effective communication in NICU every other month. Furthermore, creation of framework and tools for delivering bad news including ’Family Meeting Template’.	Training and tool	All three goals	The majority of the NICU staff found the family meeting template useful. Positive experiences from participants about the training sessions.
Mansson et al. 2019	Sweden, NICU	Non-RCT	Parents of infants born before 37 weeks gestation. Intervention mothers n = 59, fathers n = 59; control mothers n = 60, fathers n = 58. Target: hospital-based neonatal homecare nurses.	Newborn Health	Structured communication programme on supportive parent-centred communication based on the parents’ needs, and involves four dialogues, including daily information provision.	Tool	Exchange of information and creating relationship	Stress scores did not vary between the control and intervention groups. No significant differences revealed between parents in the intervention group except for one item ’not being able to feed the babies themselves’.
Margolis et al. 2018	USA, hospital	Post implementation evaluation study	OBGYN residents (n = 24).	Maternal Health	Training to improve code status discussion.	Training	Exchange of information	Residents felt more prepared to discuss code status and end of life discussions. No increase in performance.
Mash et al. 2008	South Africa, Namibia, Swaziland, four different counseling sites	Non-RCT	Nurse and lay prevention counsellors for mother-to-child-transmission (PMTCT), n = 38.	Maternal Health	3-day training in motivational interviewing.	Training	Exchange of information and inclusion of women in decision making	Heterogenous changes in proficiencies on motivational interviewing.
Maurer et al. 2019	USA, hospital	RCT	Pregnant women, intervention group n = 122, control group n = 123.	Maternal Health	Regular communication through messages/emails with information, tools to support discussions with providers.	Tool and other	Exchange of information	Women from intervention group were significantly more informed, and had more confidence in finding information. Higher chance to discuss preferences with doctor. No difference in increase in confidence to decide about care.
Mazza et al. 2020	Australia, family physician practice	RCT	Family physicians (intervention n = 18, control n = 21) and sexually active women between 16–45 years old, interested in discussing contraceptive methods (intervention n = 307, control n = 433).	Family Planning	6-hour training on structured contraceptive counselling.	Training	Exchange of information	Increase of prescription of long-active reversible contraceptives.
McLachlan et al. 2011	Australia, tertiary referral hospital and a regional hospital	Non-RCT	Midwives, pre-intervention n = 21, post-intervention n = 21.	Maternal Health	14-hours of training on women-centred care, active listening, discussing sensitive issues, non-directive problem solving and other communication skills.	Training	Exchange of information and creating relationship	Participants were more likely to feel competent at identifying women in an abusive relationship, encouraging them to talk about it, and to express feelings.
Meyer et al. 2011	USA, NICU units in different hospitals	Non-RCT	Physicians, nurses, social workers, psychologists, chaplains and medical interpreters (n = 74).	Newborn Health	6-hour training session on communication, based on relational learning.	Training	Creating relationship	Improvement of preparation, communication skills and confidence, and ability to establish relationships. Other themes were honouring the family perspective, appreciating interdisciplinary collaboration and personal connection.
Miazga et al. 2020	Canada, tertiary medical centre	Non-RCT	Pregnant women who had a previous caesarean section. Before-intervention n = 274, post-intervention n = 214.	Maternal Health	Package: (1) educational rounds for health care providers, (2) a physician−patient TOLAC (trial of labour after cesarian) discussion aid, and (3) patient-centred educational handouts, videos, and posters.	Training and tool	Exchange of information and inclusion of women in decision making	Caesarean section rate decreased. Induction of labour increased. No decreases in the rate of vaginal birth after CD or increases in the rates of uterine rupture or NICU admission.
Moore et al. 2017	Canada, different hospitals	Non-RCT	Women and/or partners at risk of premature birth at 23 weeks+0 days to 24 weeks+6 days GA. Intervention pregnancies n = 12, couples n = 8, women n = 3, father n = 1.	Maternal and Newborn Health	Modified decicion aid tool (PtDA). Additions: a palliative care card, integrated national survival data, addition of several new cards with other topics (maternal impact, quality of life).	Tool	Exchange of information and inclusion of women in decision making	Most participants would recommend this way of consultation. Decisional conflict scores lowered. Field testing demonstrated that consultations using the aid with decision coaching were feasible, reduced decisional conflict and may facilitate shared decision-making.
Morony et al. 2018	Australia, community nursing organization	Non-RCT	Nurses, n = 16.	Maternal and Newborn Health	7-week training on guided self-reflection and a Teach-Back (“interactive communication loop”) skills workshop (including role-play) specifically for tele-health.	Training	Exchange of information	Nurses reported that actively self-reflecting was useful developing "teach-back" skills and also the evaluation of the effect of these methods.
Moudi et al. 2020	Iran, delivery centres	Non-RCT	Women who gave birth at the delivery centres.	Maternal Health	Supportive communication techniques training programme.	Training	Creating relationship	Improved experiences of childbirth care services by women for tangibility, reliability, responsiveness, assurance, and empathy.
Munro et al. 2019	USA, 6 primary care and reproductive healthcare clinics	RCT	Clinical and administrative staff. Intervention group 12 clinics (n = approximately 70 staff members), control group 4 clinics.	Family Planning	Patient-targeted intervention: decision aid video + prompt card; and a provider-targeted intervention: decision aids + training.	Training and tool	Exchange of information and inclusion of women in decision making	The interventions were not systematically implemented in all settings. Participants had more confidence and felt the interventions were aligned well. Implementation challenges: novelty of interventions, need to modify workflows and to change behaviour.
Muthusamy et al. 2012	USA, NICU	RCT	Pregnant women at risk of premature delivery. Intervention n = 30, control n = 30.	Maternal Health	Written information provided before counselling including tips about questions to ask and visual aid.	Tool	Exchange of information and inclusion of women in decision making	No differences in knowledge of short-term problems, increased knowledge about long-term problems and numerical data in intervention group. Improved anxiety rates after intervention.
Nagle et al. 2008	Australia, primary health care clinics	RCT	Women in early pregnancy consulting a General Practitioner. Intervention n = 165, control n = 165. Target group: General Practitioners.	Maternal Health	Decision aid booklet for prenatal testing.	Tool	Exchange of information	Women in the intervention group were more likely to make an informed decision. The proportion of women in the intervention group with "good level of knowledge" was higher than compared to the control group. There were no differences between groups in psychological and "acceptability" outcomes.
Nassar et al. 2007	Australia, tertiary obstetric hospitals	RCT	Women with a singleton pregnancy and breech presentation. Intervention n = 102, n = 98 respectively.	Maternal Health	A decision aid about management options for breech presentation: a 24-page booklet and audio cd with information and worksheet.	Tool	Exchange of information and inclusion of women in decision making	Lower decisional conflict and increased knowledge in women, no increase in anxiety and improved satisfaction about decicion making in intervention group.
Nobili et al. 2007	Italy, academic hospital	RCT	Women who requested a pregnancy termination. Intervention n = 21, control n = 22.	Family Planning	Multicollaborative model of stuctured and women-centered counselling with 3 phases.	Other	All three goals	Intervention group increase of knowledge and positive attitude compared to control group.
O’Cathain et al. 2002	Wales, maternity units	RCT	Antenatal and postnatal groups of women. Antenatal women reaching 28 weeks’ gestation: pre-intervention n = 1386, post-intervention n = 1778. Postnatal women at 8 weeks after delivery: pre-intervention n = 1741, post-intervention n = 1547.	Maternal Health	Informed Choice leaflets: decision aids for women about 10 different decisions women face in pregnancy and childbirth to encourage their involvement in decisions.	Tool	Inclusion of women in decision making	No differences between women in intervention group in ’informed choice’. In antenatal intervention group there was a small increase of satisfaction. Only 75% of women in the intervention group reported that they were given the leaflet, indicating implementation issues.
Oka et al. 2019	Tanzania, hospital	Non-RCT	Pregnant women speaking Kiswahili, pre-intervention n = 148, post-intervention n = 170. Target population: nurses, n = 29.	Maternal Health	A job aid booklet with pictures and information on danger signs in local language; training of nurses on communication, counselling and use of the job aid (visual aid).	Training and tool	Exchange of information and creating relationship	Improved receiving of information by women of danger signs, improved impressions of nurses’ caring behaviour.
Olaiya et al. 2015	USA, women and infant clinics	Non-RCT	Smoking pregnant women (n = 71,526).	Maternal Health	Training of clinic staff about 5A counselling method.	Training	Exchange of information	Odds of quitting smoking were higher among women who attended a clinic after intervention.
Omer et al. 2008	Pakistan, field communities	Non-RCT	Women who had been pregnant or delivered.	Maternal and Newborn Health	Embraided cloths with different maternal practices and safe practices in pregnancy used in counselling about safety and maternal health (visual aid).	Tool	Exchange of information	Women in the intervention communities were more likely to attend prenatal checkups and to stop routine heavy work during pregnancy. Furthermore they more often gave colostrum to newborn babies, and maintained exclusive breastfeeding for four months.
Parham et al. 2019	USA, neonatology department at hospital	Non-RCT	Fellows, Neontatology, n = 17.	Newborn Health	7 workshops and modules on fundamental communication skills, determining goals of care, and navigating conflict during decision-making. Content decision making and bad news delivery (simulation included).	Training	Exchange of information and creating relationship	Increased self-rated confidence on communication skills.
Pelto et al. 2004	Brazil, municipal health centers	Non-RCT	Providers, intervention n = 16, control n = 14.	Maternal and Newborn Health	20-hours of training on counselling techniques.	Training	Exchange of information	Trained providers were more likely to engage in nutrition counseling and to deliver more extensive advice. They used skills to build relationship and ensure mothers would understand information.
Penticuff et al. 2005	USA, NICU	Non-RCT	Parents and their newborn infants admitted to one of the NICU study sites immediately after birth. Intervention group n = 77, control group n = 77.	Newborn Health	Structured infant progress chart and care planning meetings.	Tool	Exchange of information and inclusion of women in decision making	Increased collaboration and accuracy of parents’ understanding, fewer unrealistic concerns, less uncertainty and less decisional conflict, more satisfaction with process of decision making. No differences in satisfaction with care, relationship with provider and decisions made for their infants.
Peremans et al. 2010	Belgium, different GP practices	RCT	General practitioners, intervention empowered women n = 15, intervention computer decision support n = 15, control n = 13.	Family Planning	First intervention: standardized ’empowered’ patient who asked two additional questions about family planning. Second Intervention: computer decision support: pop up box with questions to be asked and the items to be discussed with the patient.	Tool and other	Exchange of information	Mean communication score was highest in the empowered patient group. Only a third of GPs used their computer programme during visits. There was no effect in communication scores of the computer programme versus control group.
Phillippi et al. 2016	USA, tertiary medical centre	Non-RCT	Pregnant women who need a consultation with perinatologist (intervention group n = 50, including interviews with women n = 15).	Maternal and Newborn Health	A collaborative model that facilitated simultaneous conversations with a maternal-fetal medicine specialist and a nurse-midwife.	Other	Exchange of information and inclusion of women in decision making	Women were positive regarding patient-centred care questions, they felt people where on the same page, information was clear, communication was good. Providers were also happy with collaboration. Similar perinatal outcomes in the group versus hospital averages.
Piccinini-Vallis et al. 2018	Canada, primary care practices	non-RCT	Family physicians who provide prenatal care (intervention n = 5, control n = 6), and patients (intervention n = 10, control n = 14).	Maternal Health	60-minute training session on 5A’s communication method.	Training and tool	Exchange of information and creating relationship	No differences in patient outcomes were observed.
Pimentel et al. 2018	USA, university hospital	Non-RCT	Pregnant delivering women, pre-implementation n = 101, post-implementation n = 90.	Maternal Health	Visual aid to communicate names, relevant information during labour.	Tool	Exchange of information and creating relationship	Increased knowledge of name of provider, increased satisfaction disappeared after controlling other variables.
Posner and Nakajima, 2011	Canada, university hospital	Non-RCT	OBGYN residents, pre and post intervention n = 14.	Maternal Health	2-hour workshop on disclosing an adverse event.	Training	All three goals	Significant increase in disclosure-communication skills after intervention.
Posner et al. 2012	Canada, tertiary care centre	RCT	OBGYN residents, intervention n = 7, control n = 9.	Maternal Health	Single training session with or without simulation about obstetric emergency and bad news delivery.	Training	All three goals	No differences in objective structured clinical examinations between groups.
Purcell-Jones et al. 2019	South Africa, secondary level hospitals	Non-RCT	Pregnant Xhosa women scheduled for caesarean delivery. Intervention group n = 83, control group n = 92.	Maternal Health	Informational video (visual aid) on spinal anesthesia in local (Xhosa) language used within counselling session.	Tool	Exchange of information	Decrease in the post-explanation anxiety score in intervention group. No difference in patient satisfaction scores between groups.
Quinn et al. 2019	USA, department of obstetrics and gynecology	Non-RCT	Nurses, n = 233.	(in)Fertility	Training on communication skills for oncology nurses about fertily issues.	Training	Exchange of information	Increased knowledge scores after intervention. Half of participants noted that they often or always discuss risk of infertility and fertility preservation options.
Reed et al. 2016	USA, university hospital	Non-RCT	Perinatal-neonatal medicine fellows, n = 8.	Newborn Health	3-hour simulation-based training. Content: edge of viability, redirection of care, and medical error.	Training	Exchange of information and creating relationship	Higher rates of self-reported being more comfortable with bad news delivery.
Roter et al. 2015	USA, prenatal outpatient clinics of academic hospital	RCT	Pregnant women, often with restricted literacy skills (n = 83) and clinicians (n = 17).	Maternal Health	20-minute communication skills improvement programme on a computer, designed to empower women in their communication with health professionals. Control: prenatal guide face to face educational session using booklet (tool).	Training	Exchange of information and inclusion of women in decision making	Women with literacy deficits in intervention group were verbally more active, disclosed more information and were rated as more dominant. Furthermore, clinicians in the intervention group were less dominant. For women who were more highly literate, clinicians were more patient-centred in the intervention group. For the non-literate women, there was a similar trend (non significant).
Sabnis et al. 2018	USA, NICU	Non-RCT	Neonatologists and other NICU staff (not specified how many).	Newborn Health	Regular scheduled family meetings, education, prompts and templates, accountability strengthening.	Tool and other	Exchange of information	Increased reporting of family meetings. No difference in parental satisfaction. Satisfaction of nurses and doctors increased.
Samandari et al. 2016	Guinea, family planning clinic	Post implementation evaluation study	Family planning clinic staff (nurse, midwife, family planning counsellor).	Family Planning	Training using principles of respectful and informed client interaction and specifically focusing on intimate partner violence screening and family planning counseling.	Training	Exchange of information and creating relationship	Providers felt that they were well-prepared to provide IPV screening. All women interviewed said that they would recommend the clinic to other women and indicated that adding IPV screening did not dilute the quality of care provided.
Sawyer et al. 2017	USA, neonatology department university hospital	Non-RCT	Neonatologist and neonatal fellows, n = 12.	Newborn Health	90-minute medical improvisation (simulation) workshop about antenatal counselling.	Training	Exchange of information	Improvement of self-rated counselling.
Segre et al. 2015	USA, home visit programs and obstetric care practices	RCT	Low income (minority) pregnant women and women with young children who also had a depression. Intervention n = 41, control n = 25. Target group: providers.	Maternal Health	30 to 50-minutes training on ’Listening Visits’, including collaborative problem solving and reflective listening skills.	Training	Creating relationship and inclusion of women in decision making	Intervention had significant positive effect on depressive severity, depressive symtoms and quality of life. No difference in self-reported measurement of symptoms. No dose effect relationship observed. Low dropout rate. High rating of quality.
Setubal et al. 2018	Brazil, medical school for residents training	RCT	Residents gynecology & obstetrics and pediatrics. Intervention n = 28, control n = 30.	Maternal and Newborn Health	First, a training programme for both groups on communication of bad news followed by simulation patient. After this randomnisation into training based on SPIKES strategy with video reviews or no training (control). Finally a new simulation for both groups.	Training	Exchange of information and creating relationship	There was no significant difference in the residents’ performances. The participants rated the simulation with feedback exercises highly.
Setubal et al. 2017 *secondary article belonging to Setubal 2018		Non-RCT						Residents were interested in the programme, and assessed the training as a way to systematically approach communication of bad news.
Shah et al. 2019	USA, family planning institutions	Non-RCT	Staff of family planning clinic. Pre-intervention n = 85, post intervention n = 83.	Family Planning	Clinical decision support by a screening questions prompt.	Tool	Exchange of information	High levels of comfort asking family planning service questions, increased rate of documentation.
Shao et al. 2018	China, NICU hospital	Non-RCT	NICU nursus, n = 32.	Newborn Health	Several hours simulation-based training for empathic communication skills.	Training	Creating relationship	Majority of nurses were satisfied with the training, improvement of confidence, understanding and attitude against empathy skills
Skene et al. 2019	England, NICU	Non-RCT (qualitative study)	Parents of infants at NICU (n = 80), and nurses (n = 141).	Newborn Health	Family-centred care philosophy integrated with posters, job descriptions, guidelines etc.	Tool	Creating relationship and inclusion of women in decision making	Successful implementation of interventions. Nurses and parents perceived improvement of family centred care, specifically on sharing information, providing support to families, enabling parental participations and improved caregiving by parents.
Smithbattle et al. 2013	USA, Mid-Western urban community	Non-RCT	Teenage mothers, intervention group n = 9, usual care n = 10. Target group: public health nurses (n = 6).	Maternal Health	Training focusing on empathy by using narrative methods and therapeutic tools (baby journal and therapeutic letter writing).	Training and tool	Exchange of information and creating relationship	No differences in maternal outcomes. No statistically significant difference in relationship between nurse/patient. From qualitative interviews: improved self-disclosure and fruitful dialogues, better able to look at the person in its context
Sorce et al. 2019	USA, level II hospital	Non-RCT	Nurses in the perinatal nursery, pre-intervention n = 54, post-intervention n = 54.	Newborn Health	Training including simulation on perinatal bereavement communication using a mindfulness-based bereavement care model.	Training	Exchange of information and creating relationship	Participants’ knowledge and comfort levels significantly improved after the education session. Observations during the standardized patient scenarios demonstrated that the majority of nurses used appropriate communication techniques with the bereaved mother, that was reviewed throughout the education session.
Stapleton et al. 2002	Wales/UK, maternity units	RCT	Childbearing women and health professionals who provide antenatal care. Observations n = 886, interviews n = 383.	Maternal Health	Informed Choice leaflet including training on its use.	Training and tool	Exchange of information and inclusion of women in decision making	Health professionals were positive about the leaflets and their potential. However, time pressure limited discussion, and in practice choice was often not available. Women’s trust did influence their compliance. Midwifes seldomly discussed the contents of the leaflets with women.
Stern et al. 2013	Sweden, student health clinic	RCT	Students who came for contraceptive counseling, chlamydia testing or cervical screening.	Family Planning	Semi-structured intervention with targeted information provision (about reproductive life planning) to meet women’s needs.	Tool	Exchange of information and inclusion of women in decision making	Increased knowledge of women about reproductive life planning in intervention group.
Suryavanshi et al. 2020	India, counseling and HIV testing centres in communities	RCT	Community outreach workers (intervention n = 60, control n = 56) and HIV-positive pregnant/postpartum women (intervention n = 487, control n = 397).	Maternal Health	One-week training, consisting of personal empowerment exercise, active learning techniques to practice counselling strategies, counselling scripts and education videos and M-health application.	Training and tool	Exchange of information	Higher uptake of exclusive breastfeeding at 2 months and early infant diagnosis at 6 weeks. No differences in maternal or infant deaths per group.
Tektas et al. 2017	Turkey, two academic hospitals	RCT	Pregnant women with a history of pregnancy loss. Intervention n = 55, control n = 46.	Maternal Health	A semi-structured dialogue focusing on interpersonal relationships, expressing needs of women and interpersonal care healing methods.	Tool	Creating relationship	Significant lower rates of anxiety, depression, hopelessness and prenatal attachment of women in the intervention.
Teshome et al. 2019	Ethiopia, teaching hospital	Non-RCT	Women who underwent elective or emergency obstetric or gynaecological surgeries. Intervention n = 227, control n = 230.	Maternal Health	Four-component quality improvement intervention: standard SIC (surgical informed consent) form, wall poster, training of health professionals, delivery of post-training support to professionals.	Training and tool	Exchange of information	Higher number of standard counselling components post-intervention. However, the improvement may not endure without sustained intervention.
Toivonen et al. 2020	Finland, NICUs	Non-RCT, post implementation evaluation study	Clinicians (doctors), pre-intervention n = 21 and post-intervention n = 19. Nurses of the units, pre-intervention n = 30, post-intervention n = 32. Parents, pre-intervention n = 26, post-intervention n = 36.	Newborn Health	Training programme ’Close Collaboration with Parents’, includes identification of individual needs of infants, listening to parents’ perceptions, understanding the individual story of parents, integration of parents in decision making.	Training	Creating relationship and inclusion of women in decision making	The quality of care increased significantly after the intervention in all eight units. Significant improvements in: active care by parent and staff, parent and family support, communication, developmental care, empowered decision making, facilities, guidelines and policies, staff skills and training, information provision, service improvement and parent involvement.
Toivonen et al. 2019 * secondary article belonging to Toivonen 2020			Unit managers (n = 19) and nurses (n = 32) were interviewed after implementation of the strategy.					Multidisciplinary commitment and staff motivation to change their role were key factors enabling successful implementation. Furthermore, the observable benefits were: experimental learning and the role of the mentor, support from management, correct timing.
Axelin et al. 2014 * secondary article belonging to Toivonen 2020		Post implementation evaluation study	Nurses and physicians, intervention n = 12, control n = 10.					Nurses felt the programme had promoted improved family-centred care. Increased parental involvement in infant care, more nurses’ awareness of parents’ psychosocial situation. They felt the role of the nurse changed from an "active caretaker to a facilitator" who supported parents in their care for infants.
Tsoh et al. 2010	USA, community prenatal clinics	RCT	Pregnant women, intervention n = 23, control n = 19.	Maternal Health	15-minute Video Doctor sessions plus provider cueing, The Video Doctor delivered interactive tailored messages (incl visual aids), an educational worksheet for participants and a cueing sheet for providers (suggested personalized risk communication strategies).	Tool	Exchange of information	Intervention participants were more likely to receive provider advice on tobacco use at both prenatal visits. The intervention yielded a significantly decrease in the number of days smoked and in cigarettes smoked per day.
Umbelli et al. 2015	Sudan, maternity hospital	Non-RCT	Health care providers (n = 225) and women (n = 4469).	Maternal Health	Communication training focusing on communication skills, providing support during birth, providing information and showing empathy. Duration unknown.	Training	Not able to assign	Improved information provision and improved patient satisfaction after intervention.
Verhaeghe et al. 2020	France, gynaecological emergency department, university hospital	Non-RCT	women suspected to be at risk of early pregnancy loss, pre-intervention n = 45, post-intervention n = 27. Target group: residents.	Maternal Health	3-hour simulation-based training. Content: miscarriage.	Training	Creating relationship	Lower parental grief scale after training. No differences in empathy or ’bluntness’ perceptions.
Vlemmix et al. 2015	The Netherlands, hospitals and referring midwifery practices	RCT	Pregnant women with breech presentation. Intervention group client strategy n = 562; care provider strategy n = 376; combined client and care provider strategy n = 290; controls n = 385.	Maternal Health	Client strategy (written information leaflets and decision aid); Care-provider strategy (1-day counselling course focused on knowledge and counseling skills); Combined strategy, and care as usual strategy (controls).	Training and tool	Exchange of information and inclusion of women in decision making	External version rates did not differ between groups.
Voos et al. 2011	USA, NICU	Post implementation evaluation study	Staff members (pre-intervention n = 142, post-intervention n = 136), parents (pre-intervention n = 12, post-intervention n = 16).	Newborn Health	Family Centered Rounds (FCR), where family is involved during daily rounds at NICU department.	Other	Exchange of information and inclusion of women in decision making	Increased satisfaction and collaboration after the intervention among neonatal nurses and fellows. Patients also were more satisfied about communication, meeting with physicians, and obtaining information. No differences in stress scores of parents pre- versus post-intervention.
Waisblat et al. 2017	France, public and private obstetrics institutions	Non-RCT	Women scheduled to receive epidural in labour, intervention n = 76, control n = 79. Target group of intervention: physicians.	Maternal Health	Either (a) patient rocking, gentle touching, and hypnotic communication (focus on positive suggestive communication strategies) or (b) patient rocking, gentle touching, and standard communication.	Training	Creating relationship	Reduced pain intensity and fear reported in hypnotic communication intervention group.
Weis et al. 2014	Denmark, NICU university hospital	Non-RCT	Staff nurses, n = 45.	Newborn Health	One-day training on family centred care principles: structured dialogue, reflections, and person centred communication.	Training	All three goals	In general, there were increased skills of participants.
Weis et al. 2013	Denmark, NICU at university hospital	RCT	Parents of premature infants, intervention n = 45, control n = 33.	Newborn Health	Nurse-parent intervention that aims to help parents to handle the emotional stress of parenting in a NICU unit and strengthen their ability to make decisions on infant care.	Training	Creating relationship	No differences between groups in self-reported total stress scores. No differences in parents’ self-reported experience of nurse support.
Weis et al. 2015		Post implementation evaluation study	Parents (interviews n = 22).	Newborn Health				The intervention group found scheduled dialogues and reflection sheets meaningful and supportive. scheduled dialogues took discussions to a deeper level, and were empowering. The intervention offered more structured assistance than standard care because the method guided parent-parent and parent-nurse communication to gain mutual understanding. Improved shared decision making.
Weiss et al. 2010	USA, NICU	Non-RCT	Pediatric interns, residents, neonatal fellows, attending neonatologists and neonatal nurse practitioners. Pre-intervention n = 34, post-intervention n = 50.	Newborn Health	A 30-minutes education module on family communication and a communication plan, contact card for parents and poster of health care professionals at the NICU unit.	Training and tool	Not able to assign	Higher satisfaction rates about provider communication in post-intervention group. Fewer families reported a desire for more frequent provider contact.
Whitford et al. 2014	Scotland, antenatal clinics, university or health service premises	Post implementation evaluation study	Pregnant women in their last trimester (antenatal interviews n = 42, postnatal interviews n = 29), and maternity service staff (n = 24). Target group: a range of health professionals, including midwives working in both community and hospitals, OBGYN, general practitioners providing maternity care.	Maternal Health	The use of a standard birth plan (decision aid) within a national," woman-held" maternity file.	Tool	Inclusion of women in decicion making	Participants were generally positive about the provision of the birth plan section. Perceived benefits: "the opportunity to highlight preferences, enhance communication, stimulate discussions, and address anxieties". Not all women experienced these benefits or understood the birthplan’s purpose. Health professionals recognized the need to support women with a birth plan but noted practical challenges
Wu et al. 2020	Nepal, communities	Non-RCT	Women postpartum, pre-intervention n = 445, post-intervention n = 508. Target audience: community health workers.	Family Planning	Toolkit with example questions to clarify women’s values, visual aid (and prompt). Training provider to include family members/partners.	Training and tool	Exchange of information and creating relationship	Increased use of modern contraceptive use after intervention.
Young et al. 2014	USA, tertiary university hospital	Non-RCT	OBGYN residents, n = 7.	Maternal Health	Simulation-based training about: non-judgmental communication, culture competency awareness, reflective listening.	Training	Creating relationship	Residents were satisfied with learning experiences, increased comfort levels in treating complicated patients.
Zazulak et al. 2017	USA, department of family medicine and department of obstetrics and gynecology at university hospital	non-RCT	Residents obstetrics and family medicine, intervention n = 15, control group n = 20.	Maternal Health	3-hour mindfulness and arts-based training programme to stimulate empathic responses and non-verbal communication.	Training	Creating relationship	Improvement of Mindfulness Scale domains related to self-confidence and communication. No differences between groups over the duration of the programme. Thematic analaysis: programme had positive impact on perceived empathy and the perception of personal and professional well-being.
Zethof et al. 2020	Malawi, maternity department of rural mission hospital	Non-RCT	Pregnant women undergoing caesarean section, n = 80. Target group: maternity care health workers.	Maternal Health	Provider checklist (prompt), wallposter with informed consent guide, communication training of health workers.	Training and tool	Exchange of information and inclusion of women in decision making	Recollection of informed consent for caesarean section changed significantly in the post-intervention group.

### Communication goals

Nearly all strategies (n = 126/128) addressed at least one of the communication goals (facilitating the exchange of information, creating a good interpersonal relationship, and enabling the inclusion of women and partners in the decision making [7]). Fifty-eight addressed two goals, and six studies [25–30] addressed all three goals. Box 1 provides an elaboration with examples of improvement strategies for each goal. S2 Appendix provides an overview of the communication goals of all studies.

**Box 1: Examples of improvement strategies for the three communication goals**

**Communication goal: Facilitating the exchange of information**

- Example 1. Bakker et al. 2003. Manual, intervention card and training on a counselling protocol on smoking cessation in pregnancy, consisting of 7 steps.
- Example 2. Maurer et al. 2019. Regular communication through messages/emails with information and tools to support discussions with health workers.

**Communication goal: Creating a good interpersonal relationship**

- Example 1. Shao et al. 2018. Simulation based training for NICU nurses to improve their empathic communication skills.
- Example 2. Bashour et al. 2013. Training for effective communication skills with a focus on the interaction between health workers and patients.

**Communication goal: Enabling the inclusion of women and partners/families in MNC decision making**

- Example 1. Muthusamy et al 2012. Written information to receive before counselling including tips about questions to ask.
- Example 2. Chinkam et al. 2016. Scripted counselling package about birth choices and trial of labour after caesarean using shared decision-making principles.

The goal ‘facilitating the exchange of information’ was present in most strategies (n = 98/128) [25–137]. Examples included visual aids [visuals], decision tools, and health worker training focused on the information aspect of communication.

In total, 58 studies aimed to improve the goal ‘creating a good interpersonal relationship’ (n = 58/128) [25–30, 38, 39, 73, 75, 77–79, 82–84, 90–94, 104, 106, 109, 110, 112–115, 120–122, 131, 132, 138–164]. These often sought to improve relationships by enhancing verbal and non-verbal communication, including touching the patient, showing empathy and compassion.

The goal ‘inclusion of women and partners in the decision making’ was addressed by 41 strategies (n = 41/128) [25–30, 32–35, 43–45, 69–71, 80, 81, 85, 88, 89, 95–98, 100, 102, 107, 108, 117, 134–137, 140, 141, 160–169], for example by asking women about their values and beliefs. Often a decision aid tool was used, such as the WHO Family Planning Care Guidance (FPCG) flipchart decision aid where both women and health workers have information presented on their ‘side’ of a flipchart to support provision of information and shared decision making [35, 70, 96, 170]. Training sessions were regularly used to improve this goal too, for example in Toivonen et al. (2020) [141], where health workers in a neonatal intensive care unit were trained to collaborate with parents using shared decision making and person-centred care principles.

### Strategies to improve communication

Two main types of strategy were used to improve interpersonal communication: training of health workers (n = 81) and tools to facilitate interpersonal communication (n = 67), with a few employing other distinct approaches (n = 7). Box 2 provides examples of these two main strategies.

**Box 2: Examples of the two main strategies used to improve communication**

**Training of health workers**

Training of health workers to improve their communication.

- Example 1: Toivonen et al. 2020. An education intervention to increase the quality of family-centred care in different NICU’s.
- Example 2: Posner et al. 2011. Workshop for residents in obstetrics and gynaecology on disclosing an adverse event.

**Tools to facilitate communication**

An (electronic) aid that can be used by health workers or women and partners to improve communication

- Example 1: Langston et al. 2010. WHO decision support tool to structure the family planning counselling session.
- Example 2: Kakkilaya et al. 2011. Visual aid with visual/graphical information for parents when delivery at the threshold of viability is imminent.

#### Training of health workers

The majority of studies (n = 81/128) trained health workers to improve interpersonal communication skills as a single strategy, or as one of their strategies [25, 26, 28–30, 36–42, 46, 47, 49–51, 53, 55, 56, 58, 59, 61, 63, 66, 68, 73, 77, 79–81, 83–85, 88, 90–94, 99, 104–106, 108, 110, 112–117, 120–122, 127, 128, 130, 132, 135, 137–143, 145–149, 151–153, 155–159, 161–163, 165, 168, 171, 172]. Training programmes had different durations, ranging from 30 minutes [46, 163] to several months [26]. Nineteen studies (n = 19/82) used simulation-based training to teach communication skills [29, 38–41, 47, 68, 77, 84, 90, 91, 105, 110, 112, 114, 115, 151, 153, 155, 157, 161]. Some studies employed specific underlying communication theories as a basis of their training, for example the 5-A method for counselling [46, 61, 79, 104] or motivational interviewing techniques [26, 81, 104, 121]. Often, training was combined with communication tools such as scripts or guidelines to provide guidance, structure or reminders [26, 51, 72, 79, 80, 83, 85, 88, 92, 93, 99, 104, 108, 116, 173].

#### Tools to facilitate interpersonal communication

Sixty-seven studies (n = 67/128) used tools to improve interpersonal communication between health workers and women and partners: decision aids, visual aids, prompts and scripts, and guidelines based on specific theory-based approaches to communication [25, 26, 31–33, 35–37, 43–45, 48, 50–52, 54, 57, 60, 62, 64, 65, 67, 69–71, 75, 78–80, 82, 83, 85, 87–89, 92, 93, 95–100, 102–104, 108, 109, 116, 117, 123–126, 128–132, 134–136, 144, 154, 160, 164, 166–168, 172, 174].

Decision aids (n = 23/67) [32, 33, 35, 43–45, 60, 64, 69–71, 80, 87, 88, 95–97, 108, 123, 124, 126, 134, 136, 167, 168, 174] were often used as tools to support health workers, and/or women and partners in decision making about a health-related issue. Visual aids were used in 17 strategies (n = 17/67) [36, 37, 48, 50–52, 62, 65, 75, 80, 83, 92, 103, 108, 117, 128, 135, 136], and supported health workers in their interpersonal communication and explanations. An example of a culturally sensitive visual aid was a cloth embroidery depicting safe maternal practices in pregnancy [62]. Prompts (n = 11/67) were also regularly used [26, 31, 54, 67, 85, 87, 88, 92, 123, 129, 175]. In these studies, health workers (or women [88]) received a (computer-assisted) cue or prompt to deliver or ask for counselling. A fourth type of tool used in studies was a script, or guideline regarding a specific approach to interpersonal communication often based on underlying communication theory (n = 30/67) [25, 26, 31, 54, 57, 67, 78, 79, 82, 85, 89, 92, 98–100, 102–104, 109, 116, 125, 129–132, 144, 154, 160, 164, 166]. This was primarily developed for the health worker, for example a small card with sample questions [166], a more extended script package [102], a checklist [85], or the use of Gamble’s approach to guide counselling [82].

#### Other strategies to improve interpersonal communication

Seven studies used other strategies to facilitate interpersonal communication, including facilitation, women and partner or family empowerment, and multidisciplinary consultations [27, 34, 57, 64, 67, 107, 150]. The strategy of La Rosa et al. [150] consisted of health workers wearing a white coat to increase patients’ confidence or to act as a non-verbal communication facilitator. Peremans et al. 2010 [64] aimed to improve the quality of communication for contraceptive counselling by general practitioners (GPs), who used a decision aid during contraceptive counselling or were confronted with a ‘standardized patient’ who was empowered to ask a few additional questions regarding their contraceptive options. Three of the seven studies used a multidisciplinary approach to improve interpersonal communication, with joint consultations involving various medical specialists, psychologists and/or nurses [27, 67, 107].

### Interpersonal communication effectiveness and outcomes

Table 1 includes a narrative overview of key findings of the included studies. Outcomes assessed were diverse, and ranged from health workers’ confidence levels in their communication skills, the participants’ experiences of care, behaviour change (e.g., contraceptive uptake), to impact on health outcomes. Most studies reported a positive effect on at least one of the outcomes measured. One article reported negative consequences after the use of a decision making tool at the NICU [134].

### Update of Feldman-Stewart and Brundage communication framework

**Fig 2:**
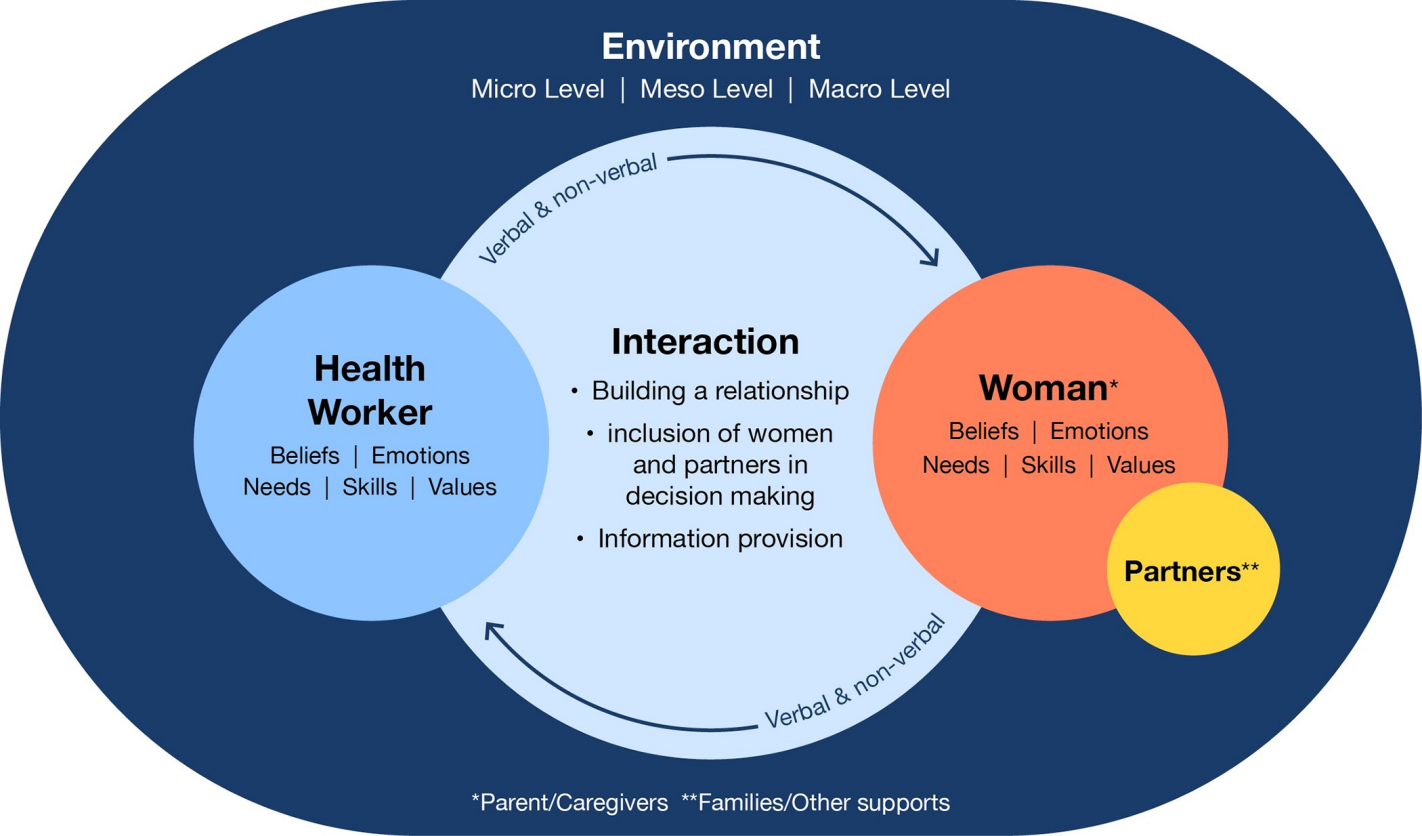
Updated framework for interpersonal communication in MNC, based on Feldman-Stewart and Brundage [14].

Based on our findings, the reflections embedded in related articles, and the reflections of the review team, we adapted the communication framework developed by Feldman-Stewart and Brundage [14] to illustrate how interpersonal communication works. We identified four ways in which this framework could be further adapted for the context of respectful MNC (Fig 2). First, we changed the name of ‘patient’ into ‘women and partners’. This is important in MNC communication because it may help remind health workers that they relate to and communicate with not just the women, but also their partners. Second, we reformulated the communication process as the *interaction* between health workers, women and partners to emphasize its bidirectional nature. This bidirectional nature was already acknowledged in the original papers for the framework by using a double arrow. By explicitly mentioning it in our updated framework we aimed to create awareness that focussing on women and their partners (as well as on the health workers) might be an important alternative strategy to improve interpersonal communication. Third, we included the three communication goals (to facilitate information exchange, create a good interpersonal relationship, and enable the inclusion of women and partners in decision making) to further explain the nature of communication processes or interaction. Including the different goals of communication may remind health workers that these three goals will need different and specific attention in case they need to be improved. Inclusion of these goals in a communication framework will facilitate making deliberate choices when designing interventions to improve interpersonal communication. Fourth, we divided the ‘environment’ into different health system levels (micro, meso, and macro) to emphasize that multiple types of context influence interpersonal communication [176]. Again, for the design of interventions these can result in a more precise conceptualization of the communication process. As such, this can facilitate a better exploration of how environmental aspects at different ‘levels’ of health systems, and the environment beyond, might influence communication.

## Discussion

This review identified 128 different interventions to improve interpersonal communication between health workers and women and partners in MNC. We found studies across different thematic areas to facilitate cross-learning for MNC. The majority were in high-income countries. They addressed three main goals of communication: facilitating the exchange of information, creating a good interpersonal relationship, and enabling the inclusion of women and partners in the decision making. The majority of studies focused on facilitation of exchange of information, and only a few incorporated all three goals. Strategies to improve interpersonal communication primarily consisted of health worker training and providing communication tools to facilitate interpersonal communication. We observed substantial heterogeneity in intervention design, implementation and outcome evaluation and measurements. This reduced the opportunities for an evaluation of effectiveness across different interventions within this scoping review.

Interpersonal communication within health care settings is a broad and diversely defined concept. Our comprehensive approach, in which we incorporated studies about interpersonal communication within pregnancy-related reproductive health domains, facilitated learning from related domains. This helped us to build on insights from other more specific reviews of interpersonal communication improvement strategies for care during antenatal care [177], and labour and childbirth [19]. Furthermore, we added value for future communication improvement activities by exploring how different communication goals were addressed. Finally, we further improved understanding of *how* interpersonal communication works within the MNC context by updating the previously developed framework. As such, in the absence of a clear definition of what ‘effective communication’ is, our classification and adapted model can contribute to developing such a clearer definition.

Although arguably the principles of patient-centred care date back to the ancient Greeks [178], the concept has recently received more attention in a push to transform health care into a more individually-tailored and rights-based approach instead of the traditional paternalistic (bio)medical model. These principles are embedded in midwifery and are related to optimal outcomes for maternal and newborn care [179]. This shift to focus on the experience of care [2] and patient-centred care [7] is reflected by the growing attention to the communication goals of ‘creating a good interpersonal relationship’ and ‘enabling the inclusion of women and families in the decision making’. However, our review found that information provision was still the dominant goal used in interventions (75%), suggesting there is scope for improvement to address the other domains relevant for patient-centred care and shared decision making. The importance of this shift to patient-centred care is illustrated by a review of women’s satisfaction with maternity care in LMICs [180]. This review demonstrated that aspects of relational communication such as listening and kindness could improve maternal satisfaction [180]. Similarly, a review on the effect of patient-provider communication on health outcomes in diverse medical settings and specialisms showed that involvement of patients in decision making could lead to improved psychological and somatic health outcomes [10].

Communication is a cornerstone of healthcare [5]. Most strategies in our review focused on health workers and their interpersonal communication skills through training or tool provision, and emphasized information provision. However, interactive communication by default includes and affects women and partners as participants, and yet only a few studies in our review specifically targeted women (and their partners) in their strategies [57, 181, 182]. Therefore, a deliberate effort to address this gap and include women, partners or newborns’ families in the design of strategies could be beneficial, especially for strategies that aim to include women and partners in decision making. Such a deliberate effort could address potential factors that affect interpersonal communication and shared decision making, such as health literacy challenges or language preferences. In addition to benefits for the individual women and partners, this can also mitigate the risk of increased health inequities that arise as a consequence of interventions that are (more) easily taken up by more wealthy, educated or literate patients [183, 184]. A number of equity and inclusion-promoting communication approaches have been previously identified. These include the use of culturally appropriate and less complex language without medical jargon, messages of short duration, and clear layouts or formats. A deliberate effort to include equity promoting approaches in communication improvement interventions presents an opportunity for health workers to engage, include and empower women and partners otherwise at (high) risk of being disengaged or marginalised, and to tackle a widening health equity gap [184–186].

Effective interpersonal communication is a core principle of respectful MNC, and all three goals of communication support this [187]. The recently documented unacceptably high number of women experiencing mistreatment and (verbal) abuse in maternity care worldwide [4, 188–191] stresses the need to implement and test strategies to improve respectful communication [189]. A recent multi-country study showed for example that many obstetric procedures were performed without the adequate informed consent of women, including caesarean section (among 10.8% of women), episiotomy (56.1%), induction of labour (26.9%) and vaginal examinations (58.9%) [4, 192]. This lack of consent could be greatly reduced by improved interpersonal communication. More generally, better interpersonal communication could lower the occurrence of mistreatment, and has been emphasized in global guidelines as a way to improve quality of care [2, 18, 193, 194] and respectful maternity care [16, 195–198].

The importance of an enabling environment, however, needs to be recognized. The health care setting at all levels (micro, meso and macro) impacts the ability of individual health workers to effectively communicate [199]. Enabling factors can include a non-excessive workload (and thus time to communicate), availability of adequate space and resources, [183, 200] and a work atmosphere where team work and good communication are the norm [199]. The enabling environment should also include the consideration of culture, which can impact understandings and expectations of what ‘good communication’ is between the health worker and women and partners [201].

This review highlights the importance of interpersonal communication between health workers and women and partners. There are other aspects of communication within MNC that can be possible anchors of quality improvement as well. These include interprofessional communication between health workers, which can be improved by simulation training [202] or ‘time outs’, deliberate interprofessional communication moments during labour [203]. Similarly, other quality improvement strategies have aimed to increase the frequency of contact moments between health workers and women, often through mobile-health [204–214]. Improved information provision by health workers can also occur without an interpersonal component, for example through an information video [215] or leaflets [216]. Finally, in addition to the targeted communication improvement strategies within the scope of this review, several successful multi-component or complex interventions have been reported, that take a comprehensive approach and target various interpersonal communication aspects simultaneously. An example is the multi-component strategy of Abuya et al. [217] which covered many respectful maternity care elements including interpersonal communication between health workers and women [217]. If, how, and in what way these intervention packages work (better), is relevant to include in future studies. Because of the complex interactions between strategies and local contexts, this requires implementation research with a learning agenda on *how* to design to make these interventions more context specific, and what the underlying mechanisms of action are.

### Strengths, limitations and future considerations

Our broad domain and systematic search enabled us to capture a large number of intervention studies and thereby to incorporate a broader perspective of effective interpersonal communication in different domains of SRH and MNH. Due to the large number of included studies, we may not have done full justice to complexities and nuances because we were only able to summarize limited information from each intervention. Language limitations may have resulted in the exclusion of relevant studies or reduced the diversity of study settings (six non-English articles were excluded). Exclusion of grey literature prevented review of potentially relevant reports from (non-governmental) organizations and other projects.

Although our search deliberately included databases that indexed journals from LMICs, the vast majority of studies were conducted in high-income settings. More research and better documentation of strategies to improve interpersonal communication in LMICs is therefore necessary, given both the need to develop culturally-tailored strategies in general and the greater health system constraints in these settings [218, 219]. Contextualized strategies appear especially relevant when targeting communication goals such as ‘building a relationship’, and ‘inclusion of patients in decision making or shared decision making’. Importantly, we believe communication strategies should *always* be adapted to local settings irrespective of their high- or low-income status. Because of the broad domain and inclusion of many studies from diverse settings, we believe our classification could serve well as a basis for designing strategies, measurement tools and implementation studies that can be further shaped and tailored to local settings.

Finally, our review also points towards the need to develop guidance for the reporting of communication interventions’ implementation and evaluation. We observed often a lack of detail on the exact design of a communication intervention and heterogeneity in reported outcomes, which reduces the opportunities for others to learn and adapt these strategies elsewhere. Such reporting guidance would ideally reflect the value of mixed methods designs to ensure evaluation studies report both *what* has been done, its *effectiveness* and an understanding of *how* the strategies worked, and whether they are sustainable over time. Existing tools [220, 221] can be used to start documenting these processes.

## Conclusion

This scoping review provides a classification of strategies to improve interpersonal communication between health workers and women and partners. This classification can be used as the foundation to inform the design and further tailoring of strategies to improve interpersonal communication, measurement tools and evaluation studies at local settings. While most communication strategies focus on the facilitation of information exchange, incorporation of the other goals of communication (creating a good interpersonal relationship, and including women and families in decision making) are essential to ensure optimal improvement of patient-centred communication in MNC. A learning agenda on how to do this especially in low-resource settings could provide concrete and actionable guidance for settings where the burden of maternal and newborn mortality is highest, and quality of care improvements are urgent.

## Supporting information

S1 Checklist PRISMA 2009 checklist. (DOC)

S1 Appendix Complete search strategy for different databases. (DOCX)

S2 Appendix Communication goals. (XLSX)
