# Supplementary material for: Strategies to improve interpersonal communication along the continuum of maternal and newborn care: A scoping review and narrative synthesis
Source: PLOS Glob Public Health. 2023 Oct 11;3(10):e0002449. doi: 10.1371/journal.pgph.0002449 (PMC10566738; doi:10.1371/journal.pgph.0002449)
Supplement: S1 Appendix — (DOCX) [file pgph.0002449.s002.docx]

**Medline/incl PUBMED**

**#1 communication**

clinical decision rules/ OR decision support techniques/ OR informed consent by minors/ OR informed consent/ OR nurse-patient relations/ OR physician-patient relations/ OR (advis* OR advic* OR communicat*  OR decision aid* OR decision making* OR decision support technique* OR diagnostic news deliver* OR patient empowerment* OR patient engagement* OR risk communication OR shared-decision making OR counsel* OR ((physician* OR clinician* OR doctor* OR nurse* OR centered OR provider* OR team) ADJ3 (patient* OR women* OR woman*) ADJ3 (relation* OR decision OR interaction))).ti,ab,kf.

**#2 healthcare providers**

birthing centers/ OR community health centers/ OR community health workers/ OR delivery rooms/ OR doulas/ OR family nurse practitioners/ OR general practitioners/ OR licensed practical nurses/ OR maternity hospitals/ OR neonatologists/ OR nurses/ OR nurse clinicians/ OR nurse midwives/ OR nurse practitioners/ OR nurse specialists/ OR nurses, community health/ OR nurses, international/ OR nurses, male/ OR nurses, neonatal/ OR nurses, pediatric/ OR nurses, public health/ OR nursing assistants/ OR pediatric nurse practitioners/ OR pediatricians/ OR physicians/ OR physicians, family/ OR physicians, primary care/ OR physicians, women/ OR  (asha OR accredited social health activist* OR lady health worker* OR barefoot doctor* OR clinical officer* OR community health aide* OR community health worker* OR doctor* OR doula* OR dula* OR family physician* OR family planning personnel OR general practitioner* OR gynaecologist* OR hca OR health assistant* OR health surveillance assistant* OR health care assistant* OR health care staff OR healthcare assistant* OR healthcare staff OR infant nurser* OR (maternity ADJ3 (worker* OR assistant* OR staff OR attendant* OR practitioner*)) OR medical staff OR resident* OR medical student* OR midwi* OR neonatologist* OR nurse* OR obstetrician* OR pediatrician* OR physician* OR birth attendant* OR village health worker* OR OB-GYN OR OBs-GYN).ti,ab,kf.

**#3 domains**

abortion, habitual/ OR abortion, missed/ OR abortion, spontaneous/ OR abortion, therapeutic/ OR abortion, induced/ OR birth setting/ OR breast feeding/ OR contraception/ OR embryo loss/ OR family planning services/ OR fertility clinics/ OR gynecology/ OR home childbirth/ OR infant care/ OR infant, extremely low birth weight/ OR infant, extremely premature/ OR infant, low birth weight/ OR infant, newborn/ OR infant, postmature/ OR infant, premature/ OR infant, small for gestational age/ OR infant, very low birth weight/ OR infant/ OR infertility, female/ OR infertility/ OR intensive care, neonatal/ OR kangaroo-mother care method/ OR labor onset/ OR labor presentation/ OR labor stage, first/ OR labor stage, second/ OR labor stage, third/ OR labor, obstetric/ OR live birth/ OR maternal health services/ OR maternal-child nursing/ OR natural childbirth/ OR neonatal nursing/ OR neonatology/ OR nurseries, infant/ OR obstetrics/ OR parturition/ OR perinatal care/ OR perinatology/ OR preconception care/ OR pregnancy in adolescence/ OR pregnancy maintenance/ OR pregnancy outcome/ OR pregnancy reduction, multifetal/ OR pregnancy, high-risk/ OR pregnancy, multiple/ OR pregnancy, quadruplet/ OR pregnancy, quintuplet/ OR pregnancy, triplet/ OR pregnancy, twin/ OR pregnancy, unplanned/ OR pregnancy, unwanted/ OR pregnancy/ OR prenatal care/ OR reproductive health services/ OR reproductive techniques/ OR safe sex/ OR sex education/ OR stillbirth/ OR term birth/ OR trial of labor/ OR (antenatal care OR ante-natal care OR birth OR breastfeeding OR breast-feeding OR childbirth OR embryoto* OR family planning OR female infertility OR fertility clinic* OR gestation OR gynecology OR infant* OR newborn* OR infertility clinic* OR intranatal OR intra-natal OR intrapartum OR intra-partum OR labor OR labour OR miscarr* OR neonat* OR obstetric* OR parturition OR perinatal care OR peri-natal care OR perinatology OR postconception fertility control OR preconception care OR pre-conception care OR pregnan* OR prenatal care OR protected sex OR reproductive health service* OR reproductive sterility OR responsible sex OR safe abortion OR safe sex OR sex education OR sterility OR subfertility OR sub-fertility OR NICU).ti,ab,kf.

**#4 patients/women**

patients/ OR child, hospitalized/ OR inpatients/ OR pregnant women/ OR women/ OR (woman OR women OR patient* OR care-receiver* OR family OR families OR mother*).ti,ab,kf.

**#5 intervention**

(clinical trials OR controlled clinical trial OR randomized controlled trial).pt. OR (intervention* OR training* OR program* OR trial* OR course OR courses OR workshop* OR random* OR control group* OR control condition* OR pretest* OR pre-test* OR posttest* OR post-test*).ti,ab,kf.

**EMBASE**

**#1 communication**

'Interpersonal communication'/de OR 'clinical decision rule'/de OR 'decision support system'/de OR 'informed consent'/de OR 'nurse patient relationship'/de OR 'doctor patient relationship'/de OR ('advis*' OR 'advic*' OR 'communicat*' OR 'decision aid*' OR 'decision making*' OR 'decision support technique*' OR 'diagnostic news deliver*' OR 'patient empowerment*' OR 'patient engagement*' OR 'risk communication' OR 'shared-decision making' OR 'counsel*' OR (('physician*' OR 'clinician*' OR 'doctor*' OR 'nurse*' OR 'centered' OR 'provider*' OR 'team') NEAR/2 ('patient*' OR 'women*' OR 'woman*') NEAR/2 ('relation*' OR 'decision' OR 'interaction'))):ti,ab,kw

**#2 healthcare providers**

'maternity ward'/de OR 'health center'/de OR 'health auxiliary'/de OR 'delivery room'/de OR 'doula'/de OR 'family nurse practitioner'/de OR 'general practitioner'/de OR 'licenced practical nurse'/de OR 'neonatologist'/de OR 'nurse'/de OR 'advanced practice nurse'/de OR 'case manager'/de OR 'expert nurse'/de OR 'first assistant'/de OR 'foreign nurse'/de OR 'male nurse'/de OR 'clinical nurse specialist'/de OR 'nurse midwife'/de OR 'nurse specialist'/de OR 'neonatal nurse'/de OR 'neonatal nurse practitioner'/de OR 'pediatric nurse'/de OR 'pediatric nurse practitioner'/de OR 'nursing assistant'/de OR 'pediatrician'/de OR 'physician'/de OR 'female physician'/de OR 'gynecologist'/de OR 'obstetrician'/de OR 'midwife'/de OR ('asha' OR 'accredited social health activist*' OR 'lady health worker*' OR 'barefoot doctor*' OR 'clinical officer*' OR 'community health aide*' OR 'community health worker*' OR 'doctor*' OR 'doula*' OR 'dula*' OR 'family physician*' OR 'family planning personnel' OR 'general practitioner*' OR 'gynaecologist*' OR 'hca' OR 'health assistant*' OR 'health surveillance assistant*' OR 'health care assistant*' OR 'health care staff' OR 'healthcare assistant*' OR 'healthcare staff' OR 'infant nurser*' OR ('maternity' NEAR/2 ('worker*' OR 'assistant*' OR 'staff' OR 'attendant*' OR 'practitioner*')) OR 'medical staff' OR 'resident*' OR 'medical student*' OR 'midwi*' OR 'neonatologist*' OR 'nurse*' OR 'obstetrician*' OR 'pediatrician*' OR 'physician*' OR 'birth attendant*' OR 'village health worker*' OR 'OB-GYN' OR 'OBs-GYN'):ti,ab,kw

**#3 domains**

'abortion'/de OR 'missed abortion'/de OR 'spontaneaous abortion'/de OR 'induced abortion'/de OR 'therapeutic abortion'/de OR 'birth setting'/de OR 'breast feeding'/de OR 'contraception'/de OR 'embryo death'/de OR 'family planning'/de OR 'fertility clinic'/de OR 'gynecology'/de OR 'home delivery'/de OR 'infant care'/de OR 'extremely low birthweight'/de OR 'prematurity'/de OR 'infant'/de OR 'low birthweight'/de OR 'newborn'/de OR 'postmaturity'/de OR 'small for date infant'/de OR 'very low birthweight'/de OR 'female infertility'/de OR 'infertility'/de OR 'newborn intensive care'/de OR 'kangaroo care'/de OR 'labor onset'/de OR 'labor stage'/de OR 'labor stage 1'/de OR 'labor stage 2'/de OR 'labor stage 3'/de OR 'labor'/de OR 'live birth'/de OR 'maternal health service'/de OR 'maternal child health care'/de OR 'natural childbirth'/de OR 'newborn nursing'/de OR 'neonatology'/de OR 'nursery'/de OR 'birth'/de OR 'perinatal care'/de OR 'perinatology'/de OR 'prepregnancy care'/de OR 'adolescent pregnancy'/de OR 'pregnancy'/de OR 'pregnancy outcome'/de OR 'high risk pregnancy'/de OR 'multiple pregnancy'/de OR 'twin pregnancy'/de OR 'triplet pregnancy'/de OR 'superfetation'/de OR 'quadruplet pregnancy'/de OR 'quintuplet pregnancy'/de OR 'unplanned pregnancy'/de OR 'unwanted pregnancy'/de OR 'prenatal care'/de OR 'reproductive procedure'/de OR 'infertility therapy'/de OR 'newborn care'/de OR 'safe sex'/de OR 'sexual education'/de OR 'stillbirth'/de OR 'term birth'/de OR 'trial of labor'/de OR 'intrapartum care'/de OR 'newborn care'/de OR ('antenatal care' OR 'ante-natal care' OR 'birth' OR 'breastfeeding' OR 'breast-feeding' OR 'childbirth' OR 'embryoto*' OR 'family planning' OR 'female infertility' OR 'fertility clinic*' OR 'gestation' OR 'gynecology' OR 'infant*' OR 'newborn*' OR 'infertility clinic*' OR 'intranatal' OR 'intra-natal' OR 'intrapartum' OR 'intra-partum' OR 'labor' OR 'labour' OR 'miscarr*' OR 'neonat*' OR 'obstetric*' OR 'parturition' OR 'perinatal care' OR 'peri-natal care' OR 'perinatology' OR 'postconception fertility control' OR 'preconception care' OR 'pre-conception care' OR 'pregnan*' OR 'prenatal care' OR 'protected sex' OR 'reproductive health service*' OR 'reproductive sterility' OR 'responsible sex' OR 'safe abortion' OR 'safe sex' OR 'sex education' OR 'sterility' OR 'subfertility' OR 'sub-fertility' OR 'NICU'):ti,ab,kw

**#4 patients**

'patient'/de OR 'hospitalized child'/de OR 'hospital patient'/de OR 'pregnant woman'/de OR 'mother'/de OR 'adolescent mother'/de OR 'expectant mother'/de OR 'female'/de OR ('woman' OR 'women' OR 'patient*' OR 'care-receiver*' OR 'family' OR 'families' OR 'mother*'):ti,ab,kw

**#5 intervention**

'Intervention study'/de OR 'nursing intervention'/de OR 'clinical trial'/de OR 'controlled clinical trial'/de OR 'randomized controlled trial'/de OR 'control'/de OR 'training'/de OR ('intervention*' OR 'training*' OR 'program*' OR 'trial*' OR 'course' OR 'courses' OR 'workshop*' OR 'random*' OR 'control group*' OR 'control condition*' OR 'pretest*' OR 'pre-test*' OR 'posttest*' OR 'post-test*'):ti,ab,kw

**CINAHL**

**#1 communication**

MH("decision support systems, clinical" OR "decision support techniques" OR "decision support systems, clinical" OR "decision support techniques" OR "consent*" OR "nurse-patient relations" OR "professional-patient relations*" OR "physician-patient relations”" OR " “decision making*" OR "decision support techniques*") OR TI("advis*" OR "advic*" OR "communicat*" OR "“decision aid*" OR "decision making*" OR "decision support technique*" OR "diagnostic news deliver*" OR "patient empowerment*" OR "patient engagement*" OR "risk communication" OR "shared-decision making" OR "counsel*" OR (("physician*" OR "clinician*" OR "doctor*" OR "nurse*" OR "centered" OR "provider*" OR "team") N2 ("patient*" OR "women*" OR "woman*") N2 ("relation*" OR "decision" OR "interaction"))) OR AB("advis*" OR "advic*" OR "communicat*" OR "“decision aid*" OR "decision making*" OR "decision support technique*" OR "diagnostic news deliver*" OR "patient empowerment*" OR "patient engagement*" OR "risk communication" OR "shared-decision making" OR "counsel*" OR (("physician*" OR "clinician*" OR "doctor*" OR "nurse*" OR "centered" OR "provider*" OR "team") N2 ("patient*" OR "women*" OR "woman*") N2 ("relation*" OR "decision" OR "interaction"))) OR KW("advis*" OR "advic*" OR "communicat*" OR "“decision aid*" OR "decision making*" OR "decision support technique*" OR "diagnostic news deliver*" OR "patient empowerment*" OR "patient engagement*" OR "risk communication" OR "shared-decision making" OR "counsel*" OR (("physician*" OR "clinician*" OR "doctor*" OR "nurse*" OR "centered" OR "provider*" OR "team") N2 ("patient*" OR "women*" OR "woman*") N2 ("relation*" OR "decision" OR "interaction")))

**#2 healthcare providers**

MH("nurses" OR "alternative birth centers" OR "community health centers" OR "community health workers" OR "physicians, family" OR "delivery rooms" OR "doulas" OR "family nurse practitioners" OR "neonatologists" OR "clinical nurse specialists" OR "nurse midwives" OR "nurse practitioners" OR "clinical nurse specialists" OR "community health nursing" OR "nurses, male" OR "neonatal nurse practitioners" OR "pediatric nurse practitioners" OR "community health nursing" OR "nursing assistants" OR "pediatricians" OR "physicians" OR "physicians, family" OR "physicians, women" OR "rural health personnel" OR "home health aides" OR "physicians" OR "doulas" OR "health personnel, unlicensed" OR "health personnel" OR "medical staff" OR "interns and residents" OR "students, medical" OR "midwives" OR "neonatologists" OR "pediatricians" OR "lay midwives" OR "rural health personnel" OR "ob-gyn nurse practitioners") OR TI("asha" OR "accredited social health activist*" OR "lady health worker*" OR "barefoot doctor*" OR "clinical officer*" OR "community health aide*" OR "community health worker*" OR "doctor*" OR "doula*" OR "dula*" OR "family physician*" OR "family planning personnel" OR "general practitioner*" OR "gynaecologist*" OR "hca" OR "health assistant*" OR "health surveillance assistant*" OR "health care assistant*" OR "health care staff" OR "healthcare assistant*" OR "healthcare staff" OR "infant nurser*" OR ("maternity" N2 ("worker*" OR "assistant*" OR "staff" OR "attendant*" OR "practitioner*")) OR "medical staff" OR "resident*" OR "medical student*" OR "midwi*" OR "neonatologist*" OR "nurse*" OR "obstetrician*" OR "pediatrician*" OR "physician*" OR "birth attendant*" OR "village health worker*" OR "ob-gyn" OR "obs-gyn") OR AB("asha" OR "accredited social health activist*" OR "lady health worker*" OR "barefoot doctor*" OR "clinical officer*" OR "community health aide*" OR "community health worker*" OR "doctor*" OR "doula*" OR "dula*" OR "family physician*" OR "family planning personnel" OR "general practitioner*" OR "gynaecologist*" OR "hca" OR "health assistant*" OR "health surveillance assistant*" OR "health care assistant*" OR "health care staff" OR "healthcare assistant*" OR "healthcare staff" OR "infant nurser*" OR ("maternity" N2 ("worker*" OR "assistant*" OR "staff" OR "attendant*" OR "practitioner*")) OR "medical staff" OR "resident*" OR "medical student*" OR "midwi*" OR "neonatologist*" OR "nurse*" OR "obstetrician*" OR "pediatrician*" OR "physician*" OR "birth attendant*" OR "village health worker*" OR "ob-gyn" OR "obs-gyn") OR KW("asha" OR "accredited social health activist*" OR "lady health worker*" OR "barefoot doctor*" OR "clinical officer*" OR "community health aide*" OR "community health worker*" OR "doctor*" OR "doula*" OR "dula*" OR "family physician*" OR "family planning personnel" OR "general practitioner*" OR "gynaecologist*" OR "hca" OR "health assistant*" OR "health surveillance assistant*" OR "health care assistant*" OR "health care staff" OR "healthcare assistant*" OR "healthcare staff" OR "infant nurser*" OR ("maternity" N2 ("worker*" OR "assistant*" OR "staff" OR "attendant*" OR "practitioner*")) OR "medical staff" OR "resident*" OR "medical student*" OR "midwi*" OR "neonatologist*" OR "nurse*" OR "obstetrician*" OR "pediatrician*" OR "physician*" OR "birth attendant*" OR "village health worker*" OR "ob-gyn" OR "obs-gyn")

**#3 domains**

MH("abortion, induced" OR "abortion, spontaneous" OR "abortion, habitual" OR "birth setting" OR "breast feeding" OR "contraception" OR "family planning" OR "home childbirth" OR "infant care" OR "infant, very low birth weight" OR "infant, low birth weight" OR "infant, premature" OR "infant, high risk" OR "infant, newborn" OR "infant, postmature" OR "infant, premature" OR "infant, small for gestational age" OR "infant" OR "infertility" OR "intensive care, neonatal" OR "kangaroo care" OR "labor stage, third" OR "labor stage, second" OR "labor stage, first" OR "labor stages" OR "labor presentation" OR "delivery, obstetric" OR "maternal health services" OR "maternal-child nursing" OR "neonatal nursing" OR "neonatal intensive care nursing" OR "neonatology" OR "perinatal care" OR "perinatology" OR "prepregnancy care" OR "pregnancy in adolescence" OR "pregnancy outcomes" OR "pregnancy reduction, multifetal" OR "pregnancy, high risk" OR "pregnancy, multiple" OR "pregnancy, quadruplet" OR "pregnancy, quintuplet" OR "pregnancy, triplet" OR "pregnancy, twin" OR "pregnancy, unplanned" OR "pregnancy, unwanted" OR "pregnancy" OR "prenatal care" OR "reproduction techniques" OR "safe sex" OR "sex education" OR "perinatal death" OR "term birth" OR "prenatal care" OR "intensive care units, neonatal") OR TI("antenatal care" OR "ante-natal care " OR "birth" OR "breastfeeding" OR "breast-feeding" OR "childbirth" OR "embryoto*" OR "family planning" OR "female infertility" OR "fertility clinic*" OR "gestation" OR "gynecology" OR "infant*" OR "newborn*" OR "infertility clinic*" OR "intranatal" OR "intra-natal" OR "intrapartum" OR "intra-partum" OR "labor" OR "labour" OR "miscarr*" OR "neonat*" OR "obstetric*" OR "parturition" OR "perinatal care" OR "peri-natal care" OR "perinatology" OR "postconception fertility control" OR "preconception care" OR "pre-conception care" OR "pregnan*" OR "prenatal care" OR "protected sex" OR "reproductive health service*" OR "reproductive sterility" OR "responsible sex" OR "safe abortion" OR "safe sex" OR "sex education" OR "sterility" OR "subfertility" OR "sub-fertility" OR "nicu") OR AB("antenatal care" OR "ante-natal care " OR "birth" OR "breastfeeding" OR "breast-feeding" OR "childbirth" OR "embryoto*" OR "family planning" OR "female infertility" OR "fertility clinic*" OR "gestation" OR "gynecology" OR "infant*" OR "newborn*" OR "infertility clinic*" OR "intranatal" OR "intra-natal" OR "intrapartum" OR "intra-partum" OR "labor" OR "labour" OR "miscarr*" OR "neonat*" OR "obstetric*" OR "parturition" OR "perinatal care" OR "peri-natal care" OR "perinatology" OR "postconception fertility control" OR "preconception care" OR "pre-conception care" OR "pregnan*" OR "prenatal care" OR "protected sex" OR "reproductive health service*" OR "reproductive sterility" OR "responsible sex" OR "safe abortion" OR "safe sex" OR "sex education" OR "sterility" OR "subfertility" OR "sub-fertility" OR "nicu") OR KW("antenatal care" OR "ante-natal care " OR "birth" OR "breastfeeding" OR "breast-feeding" OR "childbirth" OR "embryoto*" OR "family planning" OR "female infertility" OR "fertility clinic*" OR "gestation" OR "gynecology" OR "infant*" OR "newborn*" OR "infertility clinic*" OR "intranatal" OR "intra-natal" OR "intrapartum" OR "intra-partum" OR "labor" OR "labour" OR "miscarr*" OR "neonat*" OR "obstetric*" OR "parturition" OR "perinatal care" OR "peri-natal care" OR "perinatology" OR "postconception fertility control" OR "preconception care" OR "pre-conception care" OR "pregnan*" OR "prenatal care" OR "protected sex" OR "reproductive health service*" OR "reproductive sterility" OR "responsible sex" OR "safe abortion" OR "safe sex" OR "sex education" OR "sterility" OR "subfertility" OR "sub-fertility" OR "nicu")

**#4 patients/women**

MH("patients" OR "child, hospitalized" OR "inpatients" OR "expectant mothers" OR "women") OR TI("woman" OR "women" OR "patient*" OR "care-receiver*" OR "family" OR "families" OR "mother*") OR AB("woman" OR "women" OR "patient*" OR "care-receiver*" OR "family" OR "families" OR "mother*") OR KW("woman" OR "women" OR "patient*" OR "care-receiver*" OR "family" OR "families" OR "mother*")

**#5 intervention**

MH("clinical trials*" OR "randomized controlled trials*" OR "early childhood intervention" OR "early intervention*" OR "intervention trials" OR "nursing interventions" OR "program development" OR "program planning" OR "program implementation" OR "program evaluation" OR "preventive trials" OR "course evaluation" OR "control group" OR "pretest-posttest design*" OR "pretest-posttest control group design") OR TI("intervention*" OR "training*" OR "program*" OR "trial*" OR "course" OR "courses" OR "workshop*" OR "random*" OR "control group*" OR "control condition*" OR "pretest*" OR "pre-test" OR "posttest*" OR "post-test*") OR AB("intervention*" OR "training*" OR "program*" OR "trial*" OR "course" OR "courses" OR "workshop*" OR "random*" OR "control group*" OR "control condition*" OR "pretest*" OR "pre-test" OR "posttest*" OR "post-test*") OR KW("intervention*" OR "training*" OR "program*" OR "trial*" OR "course" OR "courses" OR "workshop*" OR "random*" OR "control group*" OR "control condition*" OR "pretest*" OR "pre-test" OR "posttest*" OR "post-test*")

**SCOPUS**

**#1 communication**

TITLE-ABS-KEY(advis* OR advic* OR communicat*  OR "decision aid*" OR "decision making*" OR "decision support technique*" OR "diagnostic news deliver*" OR "patient empowerment*" OR "patient engagement*" OR {risk communication} OR {shared-decision making} OR counsel* OR ((physician* OR clinician* OR doctor* OR nurse* OR centered OR provider* OR team) W/2 (patient* OR women* OR woman*) W/2 (relation* OR decision OR interaction)))

**#2 healthcare providers**

TITLE-ABS-KEY({asha} OR "accredited social health activist*" OR "lady health worker*" OR "barefoot doctor*" OR "clinical officer*" OR "community health aide*" OR "community health worker*" OR doctor* OR doula* OR dula* OR "family physician*" OR family planning personnel OR "general practitioner*" OR gynaecologist* OR {hca} OR "health assistant*" OR "health surveillance assistant*" OR "health care assistant*" OR {health care staff} OR "healthcare assistant*" OR {healthcare staff} OR "infant nurser*" OR (maternity W/2 (worker* OR assistant* OR staff OR attendant* OR practitioner*)) OR {medical staff} OR resident* OR "medical student*" OR midwi* OR neonatologist* OR nurse* OR obstetrician* OR pediatrician* OR physician* OR "birth attendant*" OR "village health worker*" OR {OB-GYN} OR {OBs-GYN})

**#3 domains**

TITLE-ABS-KEY({antenatal care} OR {ante-natal care} OR {birth} OR {breastfeeding} OR {breast-feeding} OR {childbirth} OR embryoto* OR {family planning} OR {female infertility} OR "fertility clinic*" OR {gestation} OR {gynecology} OR infant* OR newborn* OR "infertility clinic*" OR {intranatal} OR {intra-natal} OR {intrapartum} OR {intra-partum} OR {labor} OR {labour} OR miscarr* OR neonat* OR obstetric* OR {parturition} OR {perinatal care} OR {peri-natal care} OR {perinatology} OR {postconception fertility control} OR {preconception care} OR {pre-conception care} OR pregnan* OR {prenatal care} OR {protected sex} OR "reproductive health service*" OR {reproductive sterility} OR {responsible sex} OR {safe abortion} OR {safe sex} OR {sex education} OR {sterility} OR {subfertility} OR {sub-fertility} OR {NICU})

**#4 patients/women**

TITLE-ABS-KEY({woman} OR {women} OR patient* OR "care-receiver*" OR {family} OR {families} OR mother*)

**#5 intervention**

TITLE-ABS-KEY(intervention* OR training* OR program* OR trial* OR {course} OR {courses} OR workshop* OR random* OR "control group*" OR "control condition*" OR pretest* OR pre-test* OR posttest* OR post-test*)

**#6 Medline**

INDEX(medline)

**#7 Publication year**

PUBYEAR AFT 1999

**PsycINFO**

**#1 communication**

informed consent/ OR physician-patient relations/ OR communication skills/ OR (advis* OR advic* OR communicat*  OR decision aid* OR decision making* OR decision support technique* OR diagnostic news deliver* OR patient empowerment* OR patient engagement* OR risk communication OR shared-decision making OR counsel* OR ((physician* OR clinician* OR doctor* OR nurse* OR centered OR provider* OR team) ADJ3 (patient* OR women* OR woman*) ADJ3 (relation* OR decision OR interaction))).ti,ab,id.

**#2 healthcare providers**

Family physicians/ OR general practitioners/ OR midwifery/ OR nurses/ OR pediatricians/ OR gynecologists/ OR obstetricians/ OR physicians/ OR (asha OR accredited social health activist* OR lady health worker* OR barefoot doctor* OR clinical officer* OR community health aide* OR community health worker* OR doctor* OR doula* OR dula* OR family physician* OR family planning personnel OR general practitioner* OR gynaecologist* OR hca OR health assistant* OR health surveillance assistant* OR health care assistant* OR health care staff OR healthcare assistant* OR healthcare staff OR infant nurser* OR (maternity ADJ3 (worker* OR assistant* OR staff OR attendant* OR practitioner*)) OR medical staff OR resident* OR medical student* OR midwi* OR neonatologist* OR nurse* OR obstetrician* OR pediatrician* OR pediatrician* OR physician* OR birth attendant* OR village health worker* OR OB-GYN OR OBs-GYN).ti,ab,id.

**#3 domains**

induced abortion/ OR spontaneous abortion/ OR birth/ OR breast feeding/ OR birth control/ OR family planning/ OR infertility/ OR fertility/ OR fertility enhancement/ OR gynecology/ OR premature birth/ OR birth weight/ OR neonatal intensive care/ OR intrapartum period/ OR "labor (childbirth)" OR midwifery/ OR natural childbirth/ OR neonatal period/ OR obstetrics/ OR pregnancy/ OR adolescent pregnancy/ OR pregnancy outcomes/ OR prenatal care/ OR reproductive health/ OR safe sex/ OR sex education/ OR (antenatal care OR ante-natal care OR birth OR breastfeeding OR breast-feeding OR childbirth OR embryoto* OR family planning OR female infertility OR fertility clinic* OR gestation OR gynecology OR infant* OR newborn* OR infertility clinic* OR intranatal OR intra-natal OR intrapartum OR intra-partum OR labor OR labour OR miscarr* OR neonat* OR obstetric* OR parturition OR perinatal care OR peri-natal care OR perinatology OR postconception fertility control OR preconception care OR pre-conception care OR pregnan* OR prenatal care OR protected sex OR reproductive health service* OR reproductive sterility OR responsible sex OR safe abortion OR safe sex OR sex education OR sterility OR subfertility OR sub-fertility OR NICU).ti,ab,id.

**#4 patients/women**

female.po. OR patients/ OR hospitalized patients/ OR medical patients/ OR mothers/ OR adolescent mothers/ OR human females/ OR expectant mothers/ OR expectant parents/ OR (woman OR women OR patient* OR care-receiver* OR family OR families OR mother*).ti,ab,id.

**#5 intervention**

clinical trial.md. OR clinical trials/ OR randomized controlled trials/ OR randomized clinical trials/ OR intervention/ OR communication skills training/ OR social skills training/ OR childbirth training/ OR (intervention* OR training* OR program* OR trial* OR course OR courses OR workshop* OR random* OR control group* OR control condition* OR pretest* OR pre-test* OR posttest* OR post-test*).ti,ab,id.

Key: / = subject headings, ti=title, ab=abstract, id=identifier, .po.=population, .md.= methodology

**SoclNDEX**

**#1 communication**

SU.EXACT("informed consent (medical law)" OR "consent (law)" OR "nurse-patient relations" OR "patient-professional relations" OR "physician-patient relations") OR TI("advis*" OR "advic*" OR "communicat*" OR "“decision aid*" OR "decision making*" OR "decision support technique*" OR "diagnostic news deliver*" OR "patient empowerment*" OR "patient engagement*" OR "risk communication" OR "shared-decision making" OR "counsel*" OR (("physician*" OR "clinician*" OR "doctor*" OR "nurse*" OR "centered" OR "provider*" OR "team") N2 ("patient*" OR "women*" OR "woman*") N2 ("relation*" OR "decision" OR "interaction"))) OR AB("advis*" OR "advic*" OR "communicat*" OR "“decision aid*" OR "decision making*" OR "decision support technique*" OR "diagnostic news deliver*" OR "patient empowerment*" OR "patient engagement*" OR "risk communication" OR "shared-decision making" OR "counsel*" OR (("physician*" OR "clinician*" OR "doctor*" OR "nurse*" OR "centered" OR "provider*" OR "team") N2 ("patient*" OR "women*" OR "woman*") N2 ("relation*" OR "decision" OR "interaction"))) OR KW("advis*" OR "advic*" OR "communicat*" OR "“decision aid*" OR "decision making*" OR "decision support technique*" OR "diagnostic news deliver*" OR "patient empowerment*" OR "patient engagement*" OR "risk communication" OR "shared-decision making" OR "counsel*" OR (("physician*" OR "clinician*" OR "doctor*" OR "nurse*" OR "centered" OR "provider*" OR "team") N2 ("patient*" OR "women*" OR "woman*") N2 ("relation*" OR "decision" OR "interaction")))

**#2 healthcare providers**

SU.EXACT("community health services" OR "community-based family planning" OR "nursing" OR "nurses" OR "physicians") OR TI("asha" OR "accredited social health activist*" OR "lady health worker*" OR "barefoot doctor*" OR "clinical officer*" OR "community health aide*" OR "community health worker*" OR "doctor*" OR "doula*" OR "dula*" OR "family physician*" OR "family planning personnel" OR "general practitioner*" OR "gynaecologist*" OR "hca" OR "health assistant*" OR "health surveillance assistant*" OR "health care assistant*" OR "health care staff" OR "healthcare assistant*" OR "healthcare staff" OR "infant nurser*" OR ("maternity" N2 ("worker*" OR "assistant*" OR "staff" OR "attendant*" OR "practitioner*")) OR "medical staff" OR "resident*" OR "medical student*" OR "midwi*" OR "neonatologist*" OR "nurse*" OR "obstetrician*" OR "pediatrician*" OR "physician*" OR "birth attendant*" OR "village health worker*" OR "ob-gyn" OR "obs-gyn") OR AB("asha" OR "accredited social health activist*" OR "lady health worker*" OR "barefoot doctor*" OR "clinical officer*" OR "community health aide*" OR "community health worker*" OR "doctor*" OR "doula*" OR "dula*" OR "family physician*" OR "family planning personnel" OR "general practitioner*" OR "gynaecologist*" OR "hca" OR "health assistant*" OR "health surveillance assistant*" OR "health care assistant*" OR "health care staff" OR "healthcare assistant*" OR "healthcare staff" OR "infant nurser*" OR ("maternity" N2 ("worker*" OR "assistant*" OR "staff" OR "attendant*" OR "practitioner*")) OR "medical staff" OR "resident*" OR "medical student*" OR "midwi*" OR "neonatologist*" OR "nurse*" OR "obstetrician*" OR "pediatrician*" OR "physician*" OR "birth attendant*" OR "village health worker*" OR "ob-gyn" OR "obs-gyn") OR KW("asha" OR "accredited social health activist*" OR "lady health worker*" OR "barefoot doctor*" OR "clinical officer*" OR "community health aide*" OR "community health worker*" OR "doctor*" OR "doula*" OR "dula*" OR "family physician*" OR "family planning personnel" OR "general practitioner*" OR "gynaecologist*" OR "hca" OR "health assistant*" OR "health surveillance assistant*" OR "health care assistant*" OR "health care staff" OR "healthcare assistant*" OR "healthcare staff" OR "infant nurser*" OR ("maternity" N2 ("worker*" OR "assistant*" OR "staff" OR "attendant*" OR "practitioner*")) OR "medical staff" OR "resident*" OR "medical student*" OR "midwi*" OR "neonatologist*" OR "nurse*" OR "obstetrician*" OR "pediatrician*" OR "physician*" OR "birth attendant*" OR "village health worker*" OR "ob-gyn" OR "obs-gyn")

**#3 domains**

SU.EXACT("abortion" OR "therapeutic abortion" OR "breastfeeding" OR "contraception" OR "family planning services" OR "gynecology" OR "childbirth at home" OR "natural childbirth" OR "premature infants" OR "infant care" OR "infant health" OR "female infertility" OR "maternal health" OR "mother-child relationship" OR "midwifery" OR "obstetrics" OR "teenage pregnancy" OR "unwanted pregnancy" OR "unplanned pregnancy" OR "multiple pregnancy" OR "prenatal care" OR "reproductive health" OR "safe sex" OR "sex education") OR TI("antenatal care" OR "ante-natal care " OR "birth" OR "breastfeeding" OR "breast-feeding" OR "childbirth" OR "embryoto*" OR "family planning" OR "female infertility" OR "fertility clinic*" OR "gestation" OR "gynecology" OR "infant*" OR "newborn*" OR "infertility clinic*" OR "intranatal" OR "intra-natal" OR "intrapartum" OR "intra-partum" OR "labor" OR "labour" OR "miscarr*" OR "neonat*" OR "obstetric*" OR "parturition" OR "perinatal care" OR "peri-natal care" OR "perinatology" OR "postconception fertility control" OR "preconception care" OR "pre-conception care" OR "pregnan*" OR "prenatal care" OR "protected sex" OR "reproductive health service*" OR "reproductive sterility" OR "responsible sex" OR "safe abortion" OR "safe sex" OR "sex education" OR "sterility" OR "subfertility" OR "sub-fertility" OR "nicu") OR AB("antenatal care" OR "ante-natal care " OR "birth" OR "breastfeeding" OR "breast-feeding" OR "childbirth" OR "embryoto*" OR "family planning" OR "female infertility" OR "fertility clinic*" OR "gestation" OR "gynecology" OR "infant*" OR "newborn*" OR "infertility clinic*" OR "intranatal" OR "intra-natal" OR "intrapartum" OR "intra-partum" OR "labor" OR "labour" OR "miscarr*" OR "neonat*" OR "obstetric*" OR "parturition" OR "perinatal care" OR "peri-natal care" OR "perinatology" OR "postconception fertility control" OR "preconception care" OR "pre-conception care" OR "pregnan*" OR "prenatal care" OR "protected sex" OR "reproductive health service*" OR "reproductive sterility" OR "responsible sex" OR "safe abortion" OR "safe sex" OR "sex education" OR "sterility" OR "subfertility" OR "sub-fertility" OR "nicu") OR KW("antenatal care" OR "ante-natal care " OR "birth" OR "breastfeeding" OR "breast-feeding" OR "childbirth" OR "embryoto*" OR "family planning" OR "female infertility" OR "fertility clinic*" OR "gestation" OR "gynecology" OR "infant*" OR "newborn*" OR "infertility clinic*" OR "intranatal" OR "intra-natal" OR "intrapartum" OR "intra-partum" OR "labor" OR "labour" OR "miscarr*" OR "neonat*" OR "obstetric*" OR "parturition" OR "perinatal care" OR "peri-natal care" OR "perinatology" OR "postconception fertility control" OR "preconception care" OR "pre-conception care" OR "pregnan*" OR "prenatal care" OR "protected sex" OR "reproductive health service*" OR "reproductive sterility" OR "responsible sex" OR "safe abortion" OR "safe sex" OR "sex education" OR "sterility" OR "subfertility" OR "sub-fertility" OR "nicu")

**#4 patients/women**

SU.EXACT("patients" OR "hospital care of children" OR "pregnant women" OR "women") OR TI("woman" OR "women" OR "patient*" OR "care-receiver*" OR "family" OR "families" OR "mother*") OR AB("woman" OR "women" OR "patient*" OR "care-receiver*" OR "family" OR "families" OR "mother*") OR KW("woman" OR "women" OR "patient*" OR "care-receiver*" OR "family" OR "families" OR "mother*")

**#5 intervention**

SU.EXACT("clinical trials") OR TI("intervention*" OR "training*" OR "program*" OR "trial*" OR "course" OR "courses" OR "workshop*" OR "random*" OR "control group*" OR "control condition*" OR "pretest*" OR "pre-test" OR "posttest*" OR "post-test*") OR AB("intervention*" OR "training*" OR "program*" OR "trial*" OR "course" OR "courses" OR "workshop*" OR "random*" OR "control group*" OR "control condition*" OR "pretest*" OR "pre-test" OR "posttest*" OR "post-test*") OR KW("intervention*" OR "training*" OR "program*" OR "trial*" OR "course" OR "courses" OR "workshop*" OR "random*" OR "control group*" OR "control condition*" OR "pretest*" OR "pre-test" OR "posttest*" OR "post-test*")

Key: SU= subject terms, , TI=title, AB=abstract, KW=author-supplied keywords

**Anthropology PLUS**

**#1 communication**

SU ( "informed consent (law)" or "informed consent (medical law)" or "informed consent (medical law) -- anthropological aspects" or "nurse-patient relationship" or "doctor-patient relationship" ) OR TX ( ("advis*" OR "advic*" OR "communicat*" OR "“decision aid*" OR "decision making*" OR "decision support technique*" OR "diagnostic news deliver*" OR "patient empowerment*" OR "patient engagement*" OR "risk communication" OR "shared-decision making" OR "counsel*" OR (("physician*" OR "clinician*" OR "doctor*" OR "nurse*" OR "centered" OR "provider*" OR "team") N2 ("patient*" OR "women*" OR "woman*") N2 ("relation*" OR "decision" OR "interaction"))) )

**#2 healthcare providers**

SU ( "community health services" or "family planning" or "nursing" or "nursing -- anthropological aspects" or "nurses" or "physicians" ) OR TX ( ("asha" OR "accredited social health activist*" OR "lady health worker*" OR "barefoot doctor*" OR "clinical officer*" OR "community health aide*" OR "community health worker*" OR "doctor*" OR "doula*" OR "dula*" OR "family physician*" OR "family planning personnel" OR "general practitioner*" OR "gynaecologist*" OR "hca" OR "health assistant*" OR "health surveillance assistant*" OR "health care assistant*" OR "health care staff" OR "healthcare assistant*" OR "healthcare staff" OR "infant nurser*" OR ("maternity" N2 ("worker*" OR "assistant*" OR "staff" OR "attendant*" OR "practitioner*")) OR "medical staff" OR "resident*" OR "medical student*" OR "midwi*" OR "neonatologist*" OR "nurse*" OR "obstetrician*" OR "pediatrician*" OR "physician*" OR "birth attendant*" OR "village health worker*" OR "ob-gyn" OR "obs-gyn") )

**#3 domains**

SU ( "abortion" or "breastfeeding" or "contraception" or "family planning services" or "gynaecology" or "childbirth at home" or "premature infants" or "infant care" or "infant feeding" or "infant health" or "infant health services" or "maternal health" or "motherhood" or "midwifery" or "obstetrics" or "teenage pregnancy" or "unwanted pregnancy" or "unplanned pregnancy" or "multiple pregnancy" or "prenatal care" or "reproductive health" or "safe sex" ) OR TX ( ("antenatal care" OR "ante-natal care " OR "birth" OR "breastfeeding" OR "breast-feeding" OR "childbirth" OR "embryoto*" OR "family planning" OR "female infertility" OR "fertility clinic*" OR "gestation" OR "gynecology" OR "infant*" OR "newborn*" OR "infertility clinic*" OR "intranatal" OR "intra-natal" OR "intrapartum" OR "intra-partum" OR "labor" OR "labour" OR "miscarr*" OR "neonat*" OR "obstetric*" OR "parturition" OR "perinatal care" OR "peri-natal care" OR "perinatology" OR "postconception fertility control" OR "preconception care" OR "pre-conception care" OR "pregnan*" OR "prenatal care" OR "protected sex" OR "reproductive health service*" OR "reproductive sterility" OR "responsible sex" OR "safe abortion" OR "safe sex" OR "sex education" OR "sterility" OR "subfertility" OR "sub-fertility" OR "nicu") )

**#4 patients/women**

SU ( "patients" or "hospital patients" or "pregnant women" or "women" ) OR TX ( ("woman" OR "women" OR "patient*" OR "care-receiver*" OR "family" OR "families" OR "mother*") )

**#5 intervention**

SU "clinical trials" OR TX ( ("intervention*" OR "training*" OR "program*" OR "trial*" OR "course" OR "courses" OR "workshop*" OR "random*" OR "control group*" OR "control condition*" OR "pretest*" OR "pre-test" OR "posttest*" OR "post-test*") )

**Key: SU=subject terms, TX= All text fields**

**Cochrane Central Register of Controlled Trials (CENTRAL)**

**#1 communication**

("advis*" OR "advic*" OR "communicat*" OR "decision aid*" OR "decision making*" OR "decision support technique*" OR "diagnostic news deliver*" OR "patient empowerment*" OR "patient engagement*" OR "risk communication" OR "shared-decision making" OR "counsel*" OR (("physician*" OR "clinician*" OR "doctor*" OR "nurse*" OR "centered" OR "provider*" OR "team") near/3 ("patient*" OR "women*" OR "woman*") near/3 ("relation*" OR "decision" OR "interaction"))):ti,ab,kw

**#2 healthcare providers**

(“asha” OR “accredited social health activist*” OR “lady health worker*” OR “barefoot doctor*” OR “clinical officer*” OR “community health aide*” OR “community health worker*” OR “doctor*” OR “doula*” OR “dula*” OR “family physician*” OR “family planning personnel” OR “general practitioner*” OR “gynaecologist*” OR “hca” OR “health assistant*” OR “health surveillance assistant*” OR “health care assistant*” OR “health care staff” OR “healthcare assistant*” OR “healthcare staff” OR “infant nurser*” OR (“maternity” near/3 (“worker*” OR “assistant*” OR “staff” OR “attendant*” OR “practitioner*”)) OR “medical staff” OR “resident*” OR “medical student*” OR “midwi*” OR “neonatologist*” OR “nurse*” OR “obstetrician*” OR “pediatrician*” OR “physician*” OR “birth attendant*” OR “village health worker*” OR “OB-GYN” OR “OBs-GYN”):ti,ab,kw

**#3 domains**

(“antenatal care” OR “ante-natal care “ OR “birth” OR “breastfeeding” OR “breast-feeding” OR “childbirth” OR “embryoto*” OR “family planning” OR “female infertility” OR “fertility clinic*” OR “gestation” OR “gynecology” OR “infant*” OR “newborn*” OR “infertility clinic*” OR “intranatal” OR “intra-natal” OR “intrapartum” OR “intra-partum” OR “labor” OR “labour” OR “miscarr*” OR ”neonat*” OR “obstetric*” OR “parturition” OR “perinatal care” OR “peri-natal care” OR “perinatology” OR “postconception fertility control” OR “preconception care” OR “pre-conception care” OR “pregnan*” OR “prenatal care” OR “protected sex” OR “reproductive health service*” OR “reproductive sterility” OR “responsible sex” OR “safe abortion” OR “safe sex” OR “sex education” OR “sterility” OR “subfertility” OR “sub-fertility” OR “NICU”):ti,ab,kw

**#4 patients/women**

(“woman” OR “women” OR “patient*” OR “care-receiver*” OR “family” OR “families” OR “mother*”):ti,ab,kw

**#5 intervention**

(“intervention*” OR “training*” OR “program*” OR “trial*” OR “course” OR “courses” OR “workshop*” OR “random*” OR “control group*” OR “control condition*” OR “pretest*” OR “pre-test*” OR “posttest*” OR “post-test*”):ti,ab,kw

**Latin American and Caribbean Health Sciences Literature (LILACS)**

**#1 communication**

tw:((mj:(decision support systems, clinical)) OR (mj:(decision support techniques)) OR (mj:(decision support systems, clinical)) OR (mj:(decision support techniques)) OR (mj:(consent)) OR (mj:(nurse-patient relations)) OR (mj:(professional-patient relations)) OR (mj:(physician-patient relations)) OR (mj:(decision making)) OR (mj:(decision support techniques)) OR (ti:(advis)) OR (ti:(advice)) OR (ti:(communicat*)) OR (ti:(decision aid)) OR (ti:(decision making)) OR (ti:(decision support technique)) OR (ti:(diagnostic news deliver)) OR (ti:(patient empowerment)) OR (ti:(patient engagement)) OR (ti:(risk communication)) OR (ti:(shared-decision making)) OR (ti:(counsel)) OR (ti:(physician)) OR (ti:(clinician)) OR (ti:(doctor)) OR (ti:(nurse)) OR (ti:(centered)) OR (ti:(provider*)) OR (ti:(team)) OR (ti:(patient)) OR (ti:(wom*n)) OR (ti:(relation)) OR (ti:(decision)) OR (ti:(interaction)) OR (ab:(advis)) OR (ab:(advice)) OR (ab:(communicat*)) OR (ab:(decision aid)) OR (ab:(decision making)) OR (ab:(decision support technique)) OR (ab:(diagnostic news deliver)) OR (ab:(patient empowerment)) OR (ab:(patient engagement)) OR (ab:(risk communication)) OR (ab:(shared-decision making)) OR (ab:(counsel)) OR (ab:(physician)) OR (ab:(clinician)) OR (ab:(doctor)) OR (ab:(nurse)) OR (ab:(centered)) OR (ab:(provider*)) OR (ab:(team)) OR (ab:(patient)) OR (ab:(wom*n)) OR (ab:(relation)) OR (ab:(decision)) OR (ab:(interaction)))

**#2 healthcare providers**

tw:((mj:(nurses)) OR (mj:(alternative birth centers)) OR (mj:(community health centers)) OR (mj:(community health workers)) OR (mj:(physicians, family)) OR (mj:(delivery rooms)) OR (mj:(doulas)) OR (mj:(family nurse practitioners)) OR (mj:(neonatologists)) OR (mj:(clinical nurse specialists)) OR (mj:(nurse midwives)) OR (mj:(nurse practitioners)) OR (mj:(clinical nurse specialists)) OR (mj:(community health nursing)) OR (mj:(nurses, male)) OR (mj:(neonatal nurse practitioners)) OR (mj:(pediatric nurse practitioners)) OR (mj:(community health nursing)) OR (mj:(nursing assistants)) OR (mj:(pediatricians)) OR (mj:(physicians)) OR (mj:(physicians, family)) OR (mj:(physicians, women)) OR (mj:(rural health personnel)) OR (mj:(home health aides)) OR (mj:(physicians)) OR (mj:(doulas)) OR (mj:(health personnel, unlicensed)) OR (mj:(health personnel)) OR (mj:(medical staff)) OR (mj:(interns AND residents)) OR (mj:(students, medical)) OR (mj:(midwives)) OR (mj:(neonatologists)) OR (mj:(pediatricians)) OR (mj:(lay midwives)) OR (mj:(rural health personnel)) OR (mj:(ob-gyn nurse practitioners)) OR (ti:(asha)) OR (ti:(accredited social health activist)) OR (ti:(ady health worker)) OR (ti:(barefoot doctor)) OR (ti:(clinical officer)) OR (ti:(community health aide)) OR (ti:(community health worker)) OR (ti:(doctor)) OR (ti:(doula)) OR (ti:(dula)) OR (ti:(family physician)) OR (ti:(family planning personnel)) OR (ti:(general practitioner*)) OR (ti:(gynaecologist)) OR (ti:(hca)) OR (ti:(health assistant)) OR (ti:(health surveillance assistant)) OR (ti:(health care assistant)) OR (ti:(health care staff)) OR (ti:(healthcare assistant)) OR (ti:(healthcare staff)) OR (ti:(infant nurser)) OR (ti:(maternity)) OR (ti:(worker)) OR (ti:(assistant)) OR (ti:(staff)) OR (ti:(attendant)) OR (ti:(practitioner*)) OR (ti:(medical staff)) OR (ti:(resident)) OR (ti:(medical student)) OR (ti:(midwi*)) OR (ti:(neonatologist)) OR (ti:(nurse)) OR (ti:(obstetrician)) OR (ti:(pediatrician)) OR (ti:(physician)) OR (ti:(birth attendant)) OR (ti:(village health worker)) OR (ti:(ob-gyn)) OR (ti:(obs-gyn)) OR (ab:(asha)) OR (ab:(accredited social health activist)) OR (ab:(lady health worker)) OR (ab:(barefoot doctor)) OR (ab:(clinical officer)) OR (ab:(community health aide)) OR (ab:(community health worker)) OR (ab:(doctor)) OR (ab:(doula)) OR (ab:(dula)) OR (ab:(family physician)) OR (ab:(family planning personnel)) OR (ab:(general practitioner*)) OR (ab:(gynaecologist)) OR (ab:(hca)) OR (ab:(health assistant)) OR (ab:(health surveillance assistant)) OR (ab:(health care assistant)) OR (ab:(health care staff)) OR (ab:(healthcare assistant)) OR (ab:(healthcare staff)) OR (ab:(infant nurser)) OR (ab:(maternity)) OR (ab:(worker)) OR (ab:(assistant)) OR (ab:(staff)) OR (ab:(attendant)) OR (ab:(practitioner)) OR (ab:(medical staff)) OR (ab:(resident)) OR (ab:(medical student)) OR (ab:(midwi*)) OR (ab:(neonatologist)) OR (ab:(nurse)) OR (ab:(obstetrician)) OR (ab:(pediatrician)) OR (ab:(physician)) OR (ab:(birth attendant)) OR (ab:(village health worker)) OR (ab:(ob-gyn)) OR (ab:(obs-gyn)))

**#3 domains**

tw:((mj:(abortion, induced)) OR (mj:(abortion, spontaneous)) OR (mj:(abortion, habitual)) OR (mj:(birth setting)) OR (mj:(breast feeding)) OR (mj:(contraception)) OR (mj:(family planning)) OR (mj:(home childbirth)) OR (mj:(infant care)) OR (mj:(infant, very low birth weight)) OR (mj:(infant, low birth weight)) OR (mj:(infant, premature)) OR (mj:(infant, high risk)) OR (mj:(infant, newborn)) OR (mj:(infant, postmature)) OR (mj:(infant, premature)) OR (mj:(infant, small for gestational age)) OR (mj:(infant)) OR (mj:(infertility)) OR (mj:(intensive care, neonatal)) OR (mj:(kangaroo care)) OR (mj:(labor stage, third)) OR (mj:(labor stage, second)) OR (mj:(labor stage, first)) OR (mj:(labor stages)) OR (mj:(labor presentation)) OR (mj:(delivery, obstetric)) OR (mj:(maternal health services)) OR (mj:(maternal-child nursing)) OR (mj:(neonatal nursing)) OR (mj:(neonatal intensive care nursing)) OR (mj:(neonatology)) OR (mj:(perinatal care)) OR (mj:(perinatology)) OR (mj:(prepregnancy care)) OR (mj:(pregnancy in adolescence)) OR (mj:(pregnancy outcomes)) OR (mj:(pregnancy reduction, multifetal)) OR (mj:(pregnancy, high risk)) OR (mj:(pregnancy, multiple)) OR (mj:(pregnancy, quadruplet)) OR (mj:(pregnancy, quintuplet)) OR (mj:(pregnancy, triplet)) OR (mj:(pregnancy, twin)) OR (mj:(pregnancy, unplanned)) OR (mj:(pregnancy, unwanted)) OR (mj:(pregnancy)) OR (mj:(prenatal care)) OR (mj:(reproduction techniques)) OR (mj:(safe sex)) OR (mj:(sex education)) OR (mj:(perinatal death)) OR (mj:(term birth)) OR (mj:(prenatal care)) OR (mj:(intensive care units, neonatal)) OR (ti:(antenatal care)) OR (ti:(ante-natal care )) OR (ti:(birth)) OR (ti:(breastfeeding)) OR (ti:(breast-feeding)) OR (ti:(childbirth)) OR (ti:(embryoto)) OR (ti:(family planning)) OR (ti:(female infertility)) OR (ti:(fertility clinic)) OR (ti:(gestation)) OR (ti:(gynecology)) OR (ti:(infant)) OR (ti:(newborn)) OR (ti:(infertility clinic)) OR (ti:(intranatal)) OR (ti:(intra-natal)) OR (ti:(intrapartum)) OR (ti:(intra-partum)) OR (ti:(labor)) OR (ti:(labour)) OR (ti:(miscarr* )) OR (ti:(neonat*)) OR (ti:(obstetric*)) OR (ti:(parturition)) OR (ti:(perinatal care)) OR (ti:(peri-natal care)) OR (ti:(perinatology)) OR (ti:(postconception fertility control)) OR (ti:(preconception care)) OR (ti:(pre-conception care)) OR (ti:(pregnan*)) OR (ti:(prenatal care)) OR (ti:(protected sex)) OR (ti:(reproductive health service)) OR (ti:(reproductive sterility)) OR (ti:(responsible sex)) OR (ti:(safe abortion)) OR (ti:(safe sex)) OR (ti:(sex education)) OR (ti:(sterility)) OR (ti:(subfertility)) OR (ti:(sub-fertility)) OR (ti:(nicu)) OR (ab:(antenatal care)) OR (ab:(ante-natal care )) OR (ab:(birth)) OR (ab:(breastfeeding)) OR (ab:(breast-feeding)) OR (ab:(childbirth)) OR (ab:(embryoto)) OR (ab:(family planning)) OR (ab:(female infertility)) OR (ab:(fertility clinic)) OR (ab:(gestation)) OR (ab:(gynecology)) OR (ab:(infant)) OR (ab:(newborn)) OR (ab:(infertility clinic)) OR (ab:(intranatal)) OR (ab:(intra-natal)) OR (ab:(intrapartum)) OR (ab:(intra-partum)) OR (ab:(labor)) OR (ab:(labour)) OR (ab:(miscarr* )) OR (ab:(neonat*)) OR (ab:(obstetric*)) OR (ab:(parturition)) OR (ab:(perinatal care)) OR (ab:(peri-natal care)) OR (ab:(perinatology)) OR (ab:(postconception fertility control)) OR (ab:(preconception care)) OR (ab:(pre-conception care)) OR (ab:(pregnan*)) OR (ab:(prenatal care)) OR (ab:(protected sex)) OR (ab:(reproductive health service)) OR (ab:(reproductive sterility)) OR (ab:(responsible sex)) OR (ab:(safe abortion)) OR (ab:(safe sex)) OR (ab:(sex education)) OR (ab:(sterility)) OR (ab:(subfertility)) OR (ab:(sub-fertility)) OR (ab:(nicu)))

**#4 patients/women**

tw:(tw:((mj:(patients )) OR (mj:(child, hospitalized)) OR (mj:(inpatients)) OR (mj:(expectant mothers)) OR (mj:(women)) OR (ab:(wom*n)) OR (ab:(patient)) OR (ab:(care-receiver)) OR (ab:(family)) OR (ab:(families)) OR (ab:(mother)) OR (ti:(wom*n)) OR (ti:(patient)) OR (ti:(care-receiver)) OR (ti:(family)) OR (ti:(families)) OR (ti:(mother))))

**#5 intervention**

tw:((mj:(clinical trials)) OR (mj:(randomized controlled trials)) OR (mj:(early childhood intervention)) OR (mj:(early intervention)) OR (mj:(intervention trials)) OR (mj:(nursing interventions)) OR (mj:(program development)) OR (mj:(program planning)) OR (mj:(program implementation)) OR (mj:(program evaluation)) OR (mj:(preventive trials)) OR (mj:(course evaluation)) OR (mj:(control group)) OR (mj:(pretest-posttest design)) OR (mj:(pretest-posttest control group design)) OR (ti:(interventions)) OR (ti:(training)) OR (ti:(program)) OR (ti:(trial)) OR (ti:(course)) OR (ti:(courses)) OR (ti:(workshop)) OR (ti:(random)) OR (ti:(control group)) OR (ti:(control condition)) OR (ti:(pretest)) OR (ti:(pre-test)) OR (ti:(posttest)) OR (ti:(post-test)) OR (ab:(intervention)) OR (ab:(training)) OR (ab:(program)) OR (ab:(trial)) OR (ab:(course)) OR (ab:(courses)) OR (ab:(workshop)) OR (ab:(random)) OR (ab:(control group)) OR (ab:(control condition)) OR (ab:(pretest)) OR (ab:(pre-test)) OR (ab:(posttest)) OR (ab:(post-test)))

**African Journals Online (AJOL)**

“Communication”|”decision aid”|”decision making”|”risk communication”|interaction “maternal health”|”neonatal health”|“antenatal care”|“family planning”|“perinatal care”|“obstetric”|”pregnancy” |”intervention”|”training”|”program”

**Global Health Library**

**#1 communication**

("advis*" OR "advic*" OR "communicat*" OR "decision aid*" OR "decision making*" OR "decision support technique*" OR "diagnostic news deliver*" OR "patient empowerment*" OR "patient engagement*" OR "risk communication" OR "shared-decision making" OR "counsel*" OR "communicat*" OR "relation*" OR "decision" OR "interaction")

**#2 healthcare providers**

(“asha” OR “accredited social health activist*” OR “lady health worker*” OR “barefoot doctor*” OR “clinical officer*” OR “community health aide*” OR “community health worker*” OR “doctor*” OR “doula*” OR “dula*” OR “family physician*” OR “family planning personnel” OR “general practitioner*” OR “gynaecologist*” OR “hca” OR “health assistant*” OR “health surveillance assistant*” OR “health care assistant*” OR “health care staff” OR “healthcare assistant*” OR “healthcare staff” OR “infant nurser*” OR “maternity” OR “medical staff” OR “resident*” OR “medical student*” OR “midwi*” OR “neonatologist*” OR “nurse*” OR “obstetrician*” OR “pediatrician*” OR “physician*” OR “birth attendant*” OR “village health worker*” OR “OB-GYN” OR “OBs-GYN”)

**#3 domains**

(“antenatal care” OR “ante-natal care “ OR “birth” OR “breastfeeding” OR “breast-feeding” OR “childbirth” OR “embryoto*” OR “family planning” OR “female infertility” OR “fertility clinic*” OR “gestation” OR “gynecology” OR “infant*” OR “newborn*” OR “infertility clinic*” OR “intranatal” OR “intra-natal” OR “intrapartum” OR “intra-partum” OR “labor” OR “labour” OR “miscarr*” OR ”neonat*” OR “obstetric*” OR “parturition” OR “perinatal care” OR “peri-natal care” OR “perinatology” OR “postconception fertility control” OR “preconception care” OR “pre-conception care” OR “pregnan*” OR “prenatal care” OR “protected sex” OR “reproductive health service*” OR “reproductive sterility” OR “responsible sex” OR “safe abortion” OR “safe sex” OR “sex education” OR “sterility” OR “subfertility” OR “sub-fertility” OR “NICU”)

**#4 patients/women**

(“woman” OR “women” OR “patient*” OR “care-receiver*” OR “family” OR “families” OR “mother*”)

**#5 intervention**

(“intervention*” OR “training*” OR “program*” OR “trial*” OR “course” OR “courses” OR “workshop*” OR “random*” OR “control group*” OR “control condition*” OR “pretest*” OR “pre-test” OR “posttest*” OR “post-test*”)
